# Synthesis and Antitumor Activity Evaluation of Compounds Based on Toluquinol

**DOI:** 10.3390/md17090492

**Published:** 2019-08-23

**Authors:** Iván Cheng-Sánchez, José A. Torres-Vargas, Beatriz Martínez-Poveda, Guillermo A. Guerrero-Vásquez, Miguel Ángel Medina, Francisco Sarabia, Ana R. Quesada

**Affiliations:** 1 Department of Organic Chemistry, Faculty of Sciences, University of Málaga, Campus de Teatinos s/n, 29071 Málaga, Spain; 2 Department of Molecular Biology and Biochemistry, Faculty of Sciences, University of Málaga, Campus de Teatinos s/n, 29071 Málaga, Spain; 3 IBIMA (Biomedical Research Institute of Málaga), 29071 Málaga, Spain; 4 CIBER of Rare Diseases; Group U741 (CB06/07/0046), 29071 Málaga, Spain

**Keywords:** toluquinol, thymoquinone, marine hydroquinone, antitumor, natural compound analogues

## Abstract

Encouraged by the promising antitumoral, antiangiogenic, and antilymphangiogenic properties of toluquinol, a set of analogues of this natural product of marine origin was synthesized to explore and evaluate the effects of structural modifications on their cytotoxic activity. We decided to investigate the effects of the substitution of the methyl group by other groups, the introduction of a second substituent, the relative position of the substituents, and the oxidation state. A set of analogues of 2-substituted, 2,3-disubstituted, and 2,6-disubstituted derived from hydroquinone were synthesized. The results revealed that the cytotoxic activity of this family of compounds could rely on the hydroquinone/benzoquinone part of the molecule, whereas the substituents might modulate the interaction of the molecule with their targets, changing either its activity or its selectivity. The methyl group is relevant for the cytotoxicity of toluquinol, since its replacement by other groups resulted in a significant loss of activity, and in general the introduction of a second substituent, preferentially in the para position with respect to the methyl group, was well tolerated. These findings provide guidance for the design of new toluquinol analogues with potentially better pharmacological properties.

## 1. Introduction

**Figure 1:**
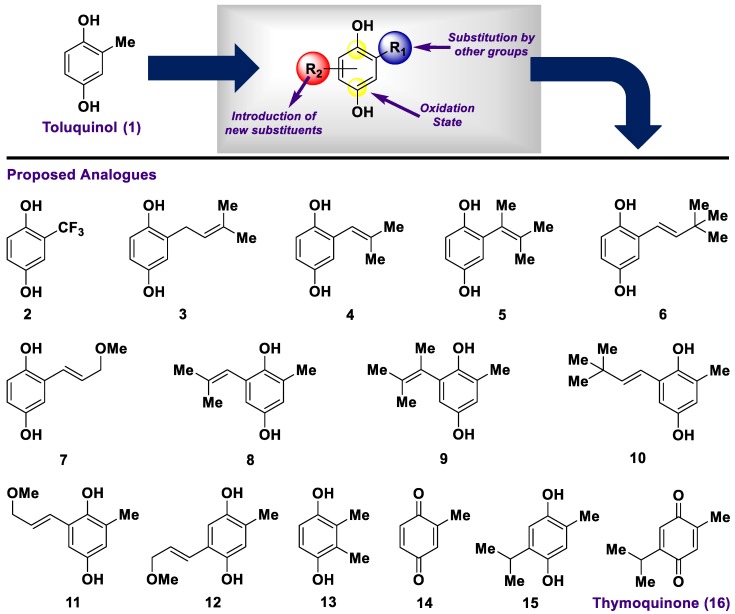
Structures of toluquinol (**1**) and programmed set of analogues **2**–**16**.

Hydroquinones and quinones are ubiquitous in nature, playing essential roles in oxidative metabolism, including in electron transport chains and photosynthesis, and are produced as primary or secondary metabolites by many organisms. A myriad of hydroquinones and quinones have been isolated from marine sources, including microbes, algae, and marine invertebrates, such as ascidians, sponges, cnidarians, and mollusks [1,2]. Some of them display a wide range of biological activities, including cytotoxicity to cancer cells, probably related to their involvement in redox cycling and/or Michael-1,4-addition reactions [3]. In many cases, the molecules responsible for the biological activities detected in some marine macroorganisms derive from the complex secondary metabolite relationship between the marine microorganisms and their hosts [4]. Marine fungi are a rich source of structurally diverse compounds with a high pharmaceutical potential [5]. In the course of our ongoing research program aimed at the search and characterization of new drug candidates of a marine origin, toluquinol (2-methylhydroquinone, compound **1**) was isolated from the culture broth of the marine fungus *Penicillium* sp. HL-85-ALS5-R004 [6]. The antitumoral, antiangiogenic, and antilymphangiogenic activities of toluquinol [6,7] make this compound a good example of the potential of marine-derived compounds in the treatment of cancer. Encouraged by these results, we decided to explore the effects of structural modifications of toluquinol on its antitumoral properties in order to establish a structure‒activity relationship as a guide to identify and develop either more potent or more specific analogues based on this natural product. These structural modifications included the substitution of the methyl group of toluquinol by different substituents (derivatives **2**–**7**), the introduction of a second substituent (derivatives **8**–**11**), the effect of the position of this second substituent (derivatives **12**–**13**), and, finally the oxidation state of the hydroquinone, such as the case of toluquinol (**1**) and toluquinone (**14**), and thymoquinol (**15**) and thymoquinone (**16**) (Figure 1).

## 2. Results and Discussion

### 2.1. Synthesis of Compounds ***2–15***

**Scheme 1:**
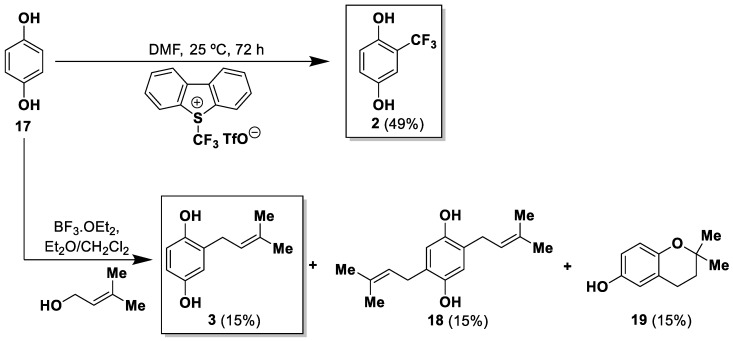
Synthesis of analogues **2** and **3**.

For the synthesis of analogues **2** and **3**, we used the procedures described in the literature [8,9], according to which the trifluoromethyl derivative **2** and the prenyl derivative **3** were prepared by direct treatment of 1,4-hydroquinone (**17**) with 5-(trifluoromethyl)-dibenzothiophenium trifluoromethanesulfonate (Umemoto’s reagent) and prenol, respectively, under acidic conditions (Scheme 1). Whereas the preparation of **2** proceeded without further issues, the synthesis of **3** was more complicated due to the low yield of the coveted prenyl derivative **3**, obtained at a poor 15% yield, and the formation of sub-products that could be identified as the disubstituted derivative **18** and the benzopyrane **19**, obtained in 3% and 2.7% yields, respectively. The three compounds obtained from this reaction could be separated by flash column chromatography and sub-products **18** and **19** were considered for inclusion in the biological studies (Scheme 1).

**Scheme 2:**
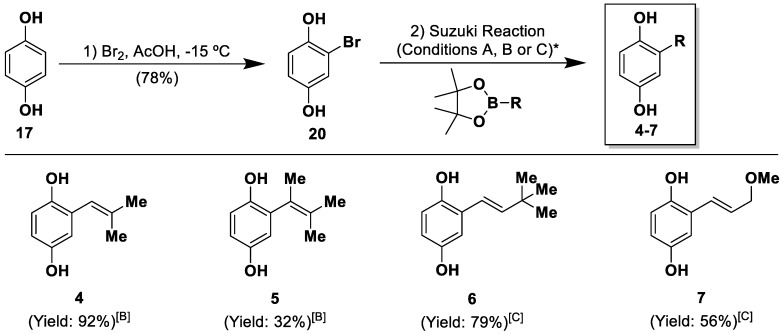
Synthesis of analogues **4**–**7** via Suzuki couplings (* Conditions A: Pd[PPh_3_]_4_, solid K_2_CO_3_, dioxane, H_2_O, 80 °C, 15 h; conditions B: Pd[PPh_3_]_4_, 2 M aq. K_2_CO_3_, THF, 80 °C, 48 h; conditions C: Pd[PPh_3_]_4_, 2 M aq. K_2_CO_3_, THF, 80 °C, 15 h).

For the synthesis of analogues **4**–**7**, we considered a Suzuki coupling reaction as a straightforward and powerful method [10] to install a vinyl-type substituent via direct coupling of the 2-bromo-1,4-hydroquinone **20**, prepared by bromination of **17 [11]**, with the corresponding pinacol boronic ester. The optimization of this coupling reaction led us to establish Pd[PPh_3_]_4_ and K_2_CO_3_ as the most suitable catalyst and base, respectively. On the other hand, the solvent, temperature, and reaction time depended on the boronic ester employed. Thus, when the reactions were undertaken using the solvent system 1,4-dioxane/H_2_O at 80 °C, which are the usual reaction conditions for Suzuki couplings (Conditions A), we observed an important degree of degradation of the reaction mixture, detecting only small amounts of the corresponding coupling products. In light of these discouraging results, we found that reactions undertaken in THF instead of 1,4-dioxane produced better results in terms of the resulting yields of the coupling products. With this new solvent system, products **4** and **5** were obtained in 92 and 32% yields, requiring a reaction time for completion of 48 h (Conditions B), and products **6** and **7** were obtained in 79 and 56% yields, respectively, after 15 h for the completion of the coupling reactions (Conditions C) (Scheme 2).

**Scheme 3:**
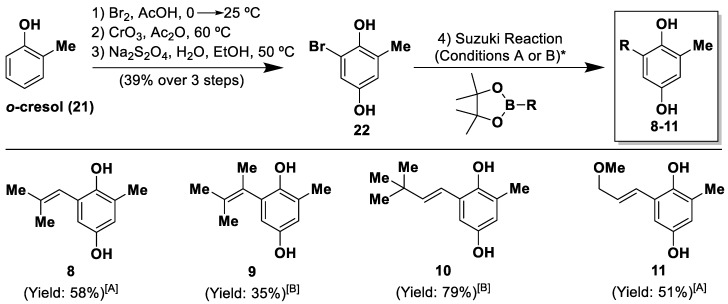
Synthesis of disubstituted analogues **8**–**11** via Suzuki couplings (* Conditions A: Pd[PPh_3_]_4_, solid K_2_CO_3_, dioxane, H_2_O, 80 °C, 15 h; conditions B: Pd[PPh_3_]_4_, 2 M aq. K_2_CO_3_, THF, 80 °C, 48 h).

For the cases of the 2,6-disubstituted 1,4-hydroquinone derivatives **8**–**11**, we devised their syntheses based again on a Suzuki coupling reaction as the most suitable method for rapid access to the targeted molecules. To this end, the bromo derivative of toluquinol **22** was prepared according to the procedures described in the literature [12] from *o*-cresol (**21**), and the corresponding Suzuki couplings were achieved following the modified conditions previously established for the synthesis of derivatives **4**–**7**. However, on this occasion we found that, for some cases (compounds **8** and **11**), the solvent system 1,4-dioxane/H_2_O provided better results compared with the use of THF as solvent, whereas for compounds **9** and **10**, THF was the solvent of choice for the Suzuki coupling (Scheme 3).

**Scheme 4:**
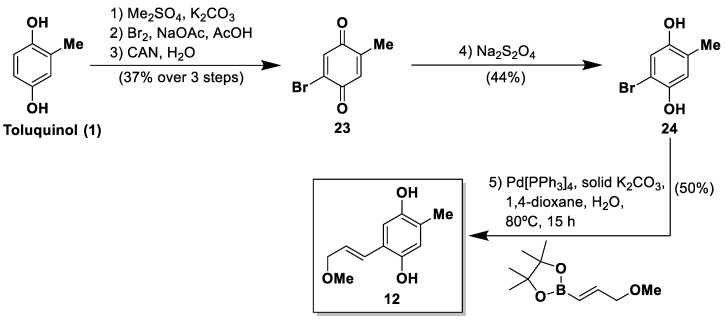
Synthesis of the *p*-disubstituted analogue **12** via a Suzuki reaction.

As a continuation of the synthetic strategy based on a Suzuki reaction for the installation of new substituents, the synthesis of the corresponding 2,5-disubstituted analogues requires the bromo derivative **24** as the key starting material. The synthesis of this bromo derivative was achieved according to the procedure described in the literature [13], starting from toluquinol (**1**) and proceeding through bromo toluquinone **23**, prepared in three steps from **1**. The reduction of this bromo quinone by the action of sodium thiosulfate provided the bromo precursor **24** at a 44% yield. The Suzuki coupling reaction of **24** with the corresponding pinacol boronic ester afforded the desired 2,5-disubstituted **12** at a reasonable 50% yield (Scheme 4).

Finally, compounds 2,3-dimethyl-1,4-hydroquinone (**13**) and toluquinone (**14**) were commercially available and, in the case of thymoquinol (**15**), its preparation was achieved from commercial thymoquinone (**16**) by reduction with sodium thiosulfate [14].

### 2.2. Antitumoral Properties of the Compounds

The synthesized 1,4-hydroquinone derivatives **2**–**12** and **15** and the commercially available compounds **13**, **14**, and **16** were subjected to biological evaluation with regard to their effect on cell growth against a panel of various human cancer cell lines. They included MDA-MB-231 (human breast adenocarcinoma), HL-60 (human promyelocytic leukemia), U87-MG (human glioblastoma), HT-1080 (human fibrosarcoma), and HT-29 (human colorectal adenocarcinoma). In these studies, toluquinol (**1**) was used as a control, for comparison purposes.

To elucidate the effect of the toluquinol analogs on the growth of tumors, logarithmically proliferating cells were treated with serial dilutions of the compounds for 72 h. Then, the number of viable cells was evaluated by the addition of MTT, and results were expressed as a percentage of viable cells versus the untreated controls. Representative dose‒response curves are presented in the Supplementary Materials. In those figures each point represents the mean of quadruplicates, and SD values were typically lower than 10% of the mean values and were omitted for clarity. The half-maximal inhibitory concentration (IC_50_) value was calculated from each dose‒response curve as the concentration of compound yielding 50% of the control cells’ survival. Table 1, containing the IC_50_ values (means ± SD of three independent experiments with quadruplicate samples each one), summarizes the results of these biological evaluations.

#### 2.2.1. Antitumor Properties with Structure‒Activity Relationship (SAR) of 2-Substituted Hydroquinones **2**–**7**

Relevant conclusions can be derived from the results presented in Table 1. At first sight, they clearly indicate a relevant role of the methyl group of toluquinol in the cytotoxic activity of this compound, since compounds lacking this methyl group (**2**–**7**) exhibited significantly increased IC_50_ values for all the tested cell lines. In this regard, substitution of the methyl group of toluquinol by a trifluoromethyl group (compound **2**) caused a 6- to 30-fold increase in the IC_50_ values for all the cell types, approaching the recently reported IC_50_ values for 1,4-hydroquinone in tumor cell lines [15]. These results are also in agreement with our previous results showing that substitution of the methyl group of toluquinol with either a carboxylic acid (as in gentisic acid) or a sulfonic acid (as in calcium dobesilate) provoked a notable decrease in the growth inhibitory activity of the compounds, as deduced from the higher IC_50_ values obtained for them in endothelial and tumor cells [6].

Many bioactive quinones and hydroquinones derived from marine organisms have one or more prenyl groups in their molecules [1,2,3]. Our results show that the substitution of a prenyl group (compound **3**) for a toluquinol methyl group practically abrogated the generic antitumor activity of the compound that only exerted a moderate cytotoxic effect against the HL60 human leukemia cell line. This activity is consistent with that already described in P388 murine leukemia cells for this natural marine compound, first isolated from the tunicate *Aplidium californicum* [16,17]. Disubstituted prenyl hydroquinones are also frequently found in marine organisms. Although a correlation between their substitution pattern and the producing organism has been proposed, there is some evidence that the pharmacophore responsible for the cytotoxicity of these naturally occurring secondary metabolites could be the quinone, hydroquinone, or naphthoquinone nucleus [1,18]. As shown in Table 1, the inclusion of a second isoprenyl group in the molecule (compound **18**, obtained as a sub-product in the synthesis of compound **3**, as shown in Scheme 1) caused a 7-fold increase in the antileukemic activity of the new compound, which remained noncytotoxic for the other tumor cell lines. This suggests that the structural modification of the natural isoprenyl hydroquinone can effectively increase its activity, maintaining the interesting putative specificity of this compound to leukemia cells.

It is worth noting that, when substituted vinyl groups were introduced instead of the methyl group, their corresponding derivatives (compounds **4**–**7**) retained some antitumor activities, with compound **7** being the most active in this series and compound **6** the least active, as an indication of the detrimental effect of the steric hindrance when either a bulky substituent is present, such as the *tert*-butyl group (compound **6**), or an increased number of methyl groups are included in the aliphatic chain (compound **5**). In any case, all of them showed significantly lower cytotoxicity than toluquinol, indicating that these structural changes are not well tolerated with regard to their antiproliferative properties and again suggesting the important role of the methyl group for the cytotoxicity of this compound.

#### 2.2.2. Antitumor Properties with SAR of 2,6-Disubstituted Hydroquinones **8**–**11**

Our results show that good tolerance was displayed after the inclusion of a second substituent in the biological properties of the resulting disubstituted 1,4-hydroquinones (compounds **8**–**13**). All those toluquinol derivatives presented antitumoral activities in the same low μM range as the original molecule. The biological evaluation of the subset comprised by the 2,6-disubstituted-1,4-hydroquinones (compounds **8**–**11**) revealed a number of intriguing findings. Although the resulting cytotoxicity values showed that the introduction of a second substituent in the toluquinol molecule was well tolerated, a significant decrease in the cytotoxic activity was only observed as the steric volume of the substituent increased (compounds **9** and **10**). This is in agreement with the abovementioned results obtained with the monosubstituted vinyl derivatives. We analyzed the effect of the position of the second substituent in the 1,4-hydroquinone.

#### 2.2.3. Antitumor Properties with SAR of 2,3-Disubstituted Hydroquinone 13 and 2,5-Disubstituted Hydroquinones **12** and **15**

A comparison of the IC_50_ values obtained for 2,3-dimethyl-1,4-hydroquinone (**13**), the 2,5-disubstituted derivatives (compounds **12** and **15**), and the 2, 6-disubstituted derivatives (compounds **8** and **11**) indicates that, although no major differences were observed when non-bulky groups were included in any of those positions, in general, 2,5-disubstituted compounds seemed to exhibit higher cytotoxic activity. In this regard, compound **12** proved to be one of the most potent analogues, exhibiting cytotoxic activity higher than that of the 2,6-disubstituted counterpart (compound **11**).

Special mention should be made of thymoquinol (compound **15**), which in our assays demonstrated very similar activity to that of toluquinol. Several thymoquinol glycosides have been isolated from plants used in folk medicine to treat infections [19,20]. The main reason that we included thymoquinol in this study is that it is the reduced form of thymoquinone (compound **16**), the major active ingredient of *Nigella sativa*, known as black seed or black cumin, which has been used as a traditional remedy to treat a variety of diseases for more than 2000 years [21]. Known as “the miracle herb of the century,” its impressive biological activities include antibacterial, antifungal, antiviral, antihelminthic, anti-inflammatory, immunomodulatory, and anticancer, as well as curative properties against cardiovascular diseases, diabetes, asthma, and oral or kidney diseases [22]. Thymoquinone, also found in many other medicinal plants, presents a wide range of biological activities of pharmacological value, including an interesting antitumor activity derived from its antiproliferative, anti-oxidant, cytotoxic, apoptogenic, and antimetastatic effects [21,23]. Thymoquinone has been recently identified as a potent anticancer compound capable of inducing apoptotic cell death by upregulating the expression of apoptotic genes p53 and caspase 9 and downregulating the expression of anti-apoptotic genes such as Bcl2 [24]. The antiangiogenic activity of thymoquinone may also contribute to enhancing the pharmacological potential of this compound in oncology [25,26].

#### 2.2.4. Antitumor Properties with SAR of Benzoquinones **14**, **16**, and Benzopyrane **19**

To shed light on the effect of the oxidation state of the toluquinol derivatives on their biological properties, we compared the activities of toluquinol (**1**) and thymoquinol (**15**) with those of their respective quinones, toluquinone (**14**) and thymoquinone (**16**). Our results show that the reduced compounds have better activities than those of their corresponding quinones, although these differences were not very significant. Although this contradicts the previous statement that the keto form of thymoquinone could be responsible for the pharmacological properties of the compound [27], it is in agreement with recent reported data showing that the reduction of thymoquinone, toluquinone, and other quinones to their respective hydroquinones increased their cytotoxic potency and selectivity on four cell lines of ovarian cancer [28]. In any case, it cannot be ruled out that both species might play a role in the toxicity of the compounds, which could generate a redox cycle in the tumor cell, responsible for the ROS production leading to cell death, in a similar mechanism to that proposed to explain the cytotoxic activities of marine terpenoquinones [3]. Supporting this hypothesis, the cytotoxic activity of thymoquinone has been related to the induction of oxidative damage in cancer cells, resulting in apoptosis [29]. Thymoquinone has a dual effect, with antioxidant properties at low concentration, but pro-oxidant ones at higher concentrations, derived from ROS generation [30]. Acting as a redox-cycler, the quinone can be metabolized by enzymatic or non-enzymatic reactions to hydroquinone or semiquinone, generating superoxide anion radicals [31]. The relevance of the cyclic interconversion of quinones and hydroquinones in the activity of these compounds might also explain the negligible cytotoxic activity of compound **19** (obtained as a subproduct in the synthesis of compound **3**, as shown in Scheme 1), in which this interconversion between the two forms is prevented.

## 3. Experimental Section

### 3.1. General Techniques

All reactions were carried out under an argon atmosphere with dry, freshly distilled solvents under anhydrous conditions, unless using aqueous reagents or otherwise noted. All solvents used in reactions were dried and distilled using standard procedures. Tetrahydrofuran (THF) was distilled from sodium benzophenone, and methylene chloride (CH_2_Cl_2_) from calcium hydride. Yields refer to chromatographically and spectroscopically (^1^H NMR) homogeneous materials, unless otherwise stated. All solutions used in workup procedures were saturated unless otherwise noted. All reagents were purchased at highest commercial quality and used without further purification unless otherwise stated. All reactions were monitored by thin-layer chromatography (TLC) using 0.25-mm silica gel plates (60F-254), using UV light (254 nm) as the visualizing agent and acidic ceric ammonium molybdate/phosphomolybdic acid or potassium permanganate solutions and heat as the developing agents. Flash column chromatography (FCC) was performed using silica gel (60 Å, particle size 230–400 mesh) under air pressure. All solvents used for chromatographic purifications were distilled prior to use. ^1^H and ^13^C NMR spectra were recorded on a Bruker DPX-400 MHz instrument (Fällanden, Switzerland) and calibrated using residual undeuterated solvent as an internal reference. Chemical shifts are reported in ppm, with the resonance resulting from incomplete deuteration of the solvent as the internal standard (^13^CDCl_3_: 7.26 ppm, s and 77.0 ppm, t; ^13^CD_3_OD: 4.87 ppm, s, 3.31 ppm, quin and 49.1 ppm, sep; ^13^C_2_D_6_OS: 2.49 ppm, quin and 39.52 ppm, sep). Data are reported as follows: chemical shift δ/ppm (multiplicity, coupling constants *J* (Hz) and integration (^1^H only)). The following abbreviations were used to explain the multiplicities: s = singlet; d = doublet; t = triplet; q = quartet; quin = quintet; b = broad; m = multiplet or combination thereof. ^13^C signals are singles, unless otherwise stated. High-resolution mass spectrometry (HRMS) was performed on a Thermo Fisher Scientific H-ESI and APCI mass spectrometer (Waltham (MA), USA) in positive mode and using an ion trap (Orbitrap) as the mass analyzer type. HRMS signals are reported to four decimal places and are within ± 5 ppm of theoretical values. Melting points were collected using a Gallenkamp melting point apparatus (London, UK), using a gradient of 0.5 °C per min.

### 3.2. Biological Material and Methods

All the cancer cell lines used in this study were obtained from the American Type Culture Collection (ATCC, Manassas, Virginia, USA). Human fibrosarcoma HT-1080 and human glioblastoma U87-MG cells were maintained in Eagle’s Minimum Essential Medium (EMEM), supplemented with 10% FBS. Human colon adenocarcinoma HT-29 cells were maintained in DMEM containing glucose (4.5 g/L) supplemented with 10% FBS. Human breast cancer carcinoma MDA-MB-231 cells were maintained in a RPMI1640 medium supplemented with 10% FBS. Acute promyelocytic leukemia HL-60 cells were maintained in a RPMI1640 medium supplemented with 20% FBS. All culture media contained glutamine (2 mM), penicillin (50 IU/mL), streptomycin (50 μg/mL), and amphotericin (1.25 μg/mL), and all cell lines were grown at 37 °C in a humidified 5% CO_2_ atmosphere.

### 3.3. Synthesis

#### 3.3.1. 2-(Trifluoromethyl)benzene-1,4-diol (**2**)

Hydroquinone **17** (50 mg, 0.45 mmol, 1.0 equiv.), 5-(Trifluoromethyl) dibenzothiophenium trifluoromethanesulfonate (362 mg, 0.9 mmol, 2.0 equiv.) and pyridine (0.04 mL, 0.45 mmol, 1.0 equiv.) were dissolved in DMF (2 mL) and the reaction mixture was stirred for 72 h at 25 °C. After this time, the reaction mixture was diluted with water and the aqueous phase was extracted with Et_2_O twice. The combined organic phases were washed sequentially with a saturated aqueous CuSO_4_ solution and brine, dried over MgSO_4_, filtered, and the solvent removed under reduced pressure. The crude mixture was purified by flash column chromatography (silica gel, 5% EtOAc in hexanes → 20% EtOAc in hexanes) to obtain **2** (39 mg, 49%) as a yellow oil [8]: R*_f_* = 0.23 (silica gel, 30% EtOAc in hexanes); ^1^H NMR (400 MHz, CD_3_OD) δ 6.88 (d, *J* = 2.9 Hz, 1 H), 6.81 (ddd, *J* = 8.8, 2.9, 0.6 Hz, 1 H), 6.76 (dd, *J* = 4.9, 4.4 Hz, 1 H); ^19^F NMR (400 MHz, CD_3_OD) δ −63.78; HRMS (H-ESI) *m*/*z*: [M + H]^+^ calculated for C_7_H_6_F_3_O_2_ 179.0320; found 179.0318.

#### 3.3.2. 2-(3-Methylbut-2-en-1-yl)benzene-1,4-diol (**3**), 2,5-bis-(3-Methylbut-2-en-1-yl)benzene-1,4-diol (**18**) and 2,2-dimethyl-6-chromanol (**19**): Reaction of **17** with Prenol

Hydroquinone **17** (300 mg, 2.72 mmol, 1.0 equiv.) and 3-methyl-2-buten-1-ol (0.55 mL, 5.45 mmol, 2.0 equiv.) were dissolved in a 1:1 mixture of Et_2_O:CH_2_Cl_2_ (10 mL). Then, BF_3_•OEt_2_ (0.1 mL, 0.54 mmol, 0.2 equiv.) was added at 0 °C and the reaction mixture was stirred for 48 h at 25 °C. After this time, cold water was added and the aqueous phase was extracted with EtOAc. The organic phase was washed with brine, dried over MgSO_4_, filtered, and the solvent removed under reduced pressure. The residue was purified by flash column chromatography (silica gel, 15% EtOAc in hexanes → 25% EtOAc in hexanes) to obtain **3** (72 mg, 15%), together with side products **18** (21 mg, 3%) and **19** (13 mg, 2.7%) [9]. [3]: white solid, R*_f_* = 0.30 (silica gel, 50% EtOAc in hexanes); m.p. = 101–103 °C; ^1^H NMR (400 MHz, CDCl_3_) δ 6.68 (d, *J* = 8.3 Hz, 1 H), 6.63–6.54 (m, 2 H), 5.29 (t, *J* = 7.3 Hz, 1 H), 3.29 (d, *J* = 7.0 Hz, 2 H), 1.77 (s, 3 H), 1.76 (s, 3 H); ^13^C NMR (100 MHz, CDCl_3_) δ 149.7, 147.5, 131.6, 128.8, 122.6, 115.7, 115.1, 112.4, 27.8, 24.5, 16.4; HRMS (H-ESI) *m*/*z*: [M + H]^+^ calculated for C_11_H_15_O_2_ 179.1072; found 179.1064. [18]: white solid; ^1^H NMR (400 MHz, CDCl_3_) δ 6.70–6.45 (m, 4 H), 5.29 (t, *J* = 7.9 Hz, 1 H), 3.31–3.24 (m, 1 H), 2.71 (dd, *J* = 13.0, 6.4 Hz, 1 H), 1.82–1.69 (m, 12 H); ^13^C NMR (100 MHz, CDCl_3_) δ 147.2, 131.4, 125.8, 122.9, 115.6, 27.5, 24.6, 16.5; HRMS (H-ESI) *m*/*z*: [M + H]^+^ calculated for C_16_H_23_O_2_ 247.1698; found 247.1708. [19]: red oil; ^1^H NMR (400 MHz, CDCl_3_) δ 6.65 (d, *J* = 8.6 Hz, 1 H), 6.62–6.53 (m, 2 H), 2.72 (t, *J* = 6.8 Hz, 2 H), 1.77 (t, *J* = 6.8 Hz, 2 H), 1.31 (s, 6 H); ^13^C NMR (100 MHz, CDCl_3_) δ 149.8, 146.9, 121.3, 117.1, 114.8, 113.9, 73.2, 32.5, 25.6, 22.2; HRMS (H-ESI) *m*/*z*: [M + H]^+^ calculated for C_11_H_15_O_2_ 179.1072; found 179.1071.

#### 3.3.3. Synthesis of Compounds **20** and **22**

2-Bromobenzene-1,4-diol (**20**). Bromine (0.56 mL, 10.90 mmol, 1.2 equiv.) was added dropwise to a solution of 1,4-hydroquinone (**17**) (1.0 g, 9.08 mmol, 1.0 equiv.) in *^t^*BuOMe (20 mL) at ‒15 °C and the reaction mixture was stirred for 2 h. After this time, the reaction mixture was quenched by the addition of 10% aqueous sodium thiosulfate solution and extracted with Et_2_O twice. The combined organic phases were washed with brine, dried over MgSO_4_, filtered, and the solvent removed under reduced pressure. The residue was purified by flash column chromatography (silica gel, 10% EtOAc in hexanes) to obtain **20** (1.3 g, 78%) as a colorless solid [11]: R*_f_* = 0.35 (silica gel, 30% EtOAc in hexanes); ^1^H NMR (400 MHz, CD_3_OD) δ 6.89 (d, *J* = 2.8 Hz, 1 H), 6.72 (d, *J* = 8.7 Hz, 1 H), 6.60 (dd, *J* = 8.7, 2.9 Hz, 1 H); ^13^C NMR (100 MHz, CD_3_OD) δ 152.0, 148.2, 120.3, 117.7, 116.4, 110.7.

2-Bromo-6-methylbenzene-1,4-diol (**22**). Bromine (2.8 mL, 55.48 mmol, 2.0 equiv.) was added dropwise over 15 min to a solution of *o*-cresol (**21**) (2.9 mL, 27.74 mmol, 1.0 equiv.) in AcOH (30 mL) at 0 °C and the reaction mixture was stirred at 25 °C for 1 h. After this time, cold water was added and the resulting white precipitate was filtered and washed with cold water to obtain 2,4-dibromo-6-methyl phenol (S1) (5.8 g, 80%) as a colorless solid that did not require further purification [12]: R*_f_* = 0.60 (20% EtOAc in hexanes); m.p. = 56–57 °C; ^1^H NMR (400 MHz, CDCl_3_) δ 7.43 (dd, *J* = 2.3, 0.6 Hz, 1 H), 7.21–7.19 (m, 1 H), 2.27 (t, *J* = 0.7 Hz, 3 H); ^13^C NMR (100 MHz, CDCl_3_) δ 149.8, 133.1, 131.3, 127.7, 112.0, 110.4, 16.5. A solution of CrO_3_ (1.5 g, 15.31 mmol, 1.1 equiv.) in H_2_O (4 mL) was added to a solution of 2,4-dibromo-6-methyl phenol (3.7 g, 13.91 mmol, 1.0 equiv.) in Ac_2_O (7 mL)/CH_3_CN (3.5 mL) and the resulting mixture was heated at 60 °C for 1.5 h. After this time, the reaction mixture was cooled to 25 °C and diluted with water. Then, the aqueous phase was extracted with CH_2_Cl_2_ three times, and the combined organic phases were washed with brine, dried over MgSO_4_, filtered, and the solvent removed under reduced pressure to obtain 2-bromo-6-methyl-*p*-benzoquinone (**S2**) (2.3 g, 85%) as an orange solid that did not require further purification: R*_f_* = 0.45 (silica gel, 20% EtOAc in hexanes); m.p. = 92–93 °C; ^1^H NMR (400 MHz, CDCl_3_) δ 7.23 (d, *J* = 2.4 Hz, 1 H), 6.65 (dq, *J* = 2.4, 1.6 Hz, 1 H), 2.14 (d, *J* = 1.6 Hz, 3 H); ^13^C NMR (100 MHz, CDCl_3_) δ 184.8, 179.9, 145.7, 138.1, 137.3, 133.4, 16.8. The resulting *p*-benzoquinone derivative (1.0 g, 4.97 mmol, 1.0 equiv.) was dissolved in a mixture of EtOH (10 mL)/H_2_O (2 mL) and the mixture was heated at 50 °C. Then, Na_2_S_2_O_4_ (1 g, 5.97 mmol, 1.2 equiv.) was added and the reaction mixture was heated at 50 °C for 1.5 h. After this time, EtOH was removed under reduced pressure, the crude mixture was diluted with water, and the aqueous phase was extracted with EtOAc. The organic phase was washed with brine, dried over MgSO_4_, filtered, and the solvent removed under reduced pressure to obtain a solid that was washed with cold CH_2_Cl_2_ to obtain **22** (585 mg, 57%) as a pale yellow solid that did not require further purification: R*_f_* = 0.25 (silica gel, 20% EtOAc in hexanes); ^1^H NMR (400 MHz, CDCl_3_) δ 6.82 (dd, *J* = 2.9, 0.5 Hz, 1 H), 6.60 (dd, *J* = 2.9, 0.7 Hz, 1 H), 2.25 (t, *J* = 0.5 Hz, 3 H); ^13^C NMR (100 MHz, CDCl_3_) δ 148.8, 144.8, 126.5, 117.7, 115.7, 109.6, 16.9; HRMS (H-ESI) *m*/*z*: [M + H]^+^ calculated for C_7_H_8_BrO_2_ 202.9708; found 202.9726.

#### 3.3.4. General Procedures for Suzuki Couplings: Synthesis of the 2-Substituted and 2,6-Disubstituted Hydroquinones **4**–**11**

Condition A: To a solution of boronic acid pinacol ester derivative (2.0 equiv.), bromo derivative (1.0 equiv.), Pd[PPh_3_]_4_ (0.2 equiv.), and K_2_CO_3_ (3.6 equiv.) in 1,4-dioxane was added a drop of water and the reaction mixture was heated at 80 °C for 15 h. After this time, a saturated aqueous NH_4_Cl solution was added and the aqueous phase was extracted with EtOAc three times. The combined organic phases were washed with brine, dried over MgSO_4_, filtered, and the solvent removed under reduced pressure. The residue was purified by flash column chromatography (silica gel) to obtain the corresponding product. Condition B: Boronic acid pinacol ester derivative (1.1 equiv.), bromo derivative (1.0 equiv.), Pd[PPh_3_]_4_ (0.05 equiv.), and K_2_CO_3_ (2.0 M aqueous solution, 6.6 equiv.) were dissolved in THF and the reaction mixture was heated at 80 °C for 48 h. After this time, a saturated aqueous NH_4_Cl solution was added and the aqueous phase was extracted with EtOAc three times. The combined organic phases were washed with brine, dried over MgSO_4_, filtered, and the solvent removed under reduced pressure. The residue was purified by flash column chromatography (silica gel) to obtain the corresponding product. Conditions C: Boronic acid pinacol ester derivative (1.1 equiv.), bromo derivative (1.0 equiv.), Pd[PPh_3_]_4_ (0.05 equiv.), and K_2_CO_3_ (2.0 M aqueous solution, 6.6 equiv.) were dissolved in THF and the reaction mixture was heated at 80 °C for 15 h. After this time, a saturated aqueous NH_4_Cl solution was added and the aqueous phase was extracted with EtOAc three times. The combined organic phases were washed with brine, dried over MgSO_4_, filtered, and the solvent removed under reduced pressure. The residue was purified by flash column chromatography (silica gel) to obtain the corresponding product.

2-(2-Methylprop-1-en-1-yl)benzene-1,4-diol (**4**). Compound **4** was prepared from bromohydroquinone **20** (50 mg, 0.29 mmol, 1.0 equiv.) by treatment with 2-methyl-1-propenylboronic acid pinacol ester (0.06 mL, 0.29 mmol, 1.1 equiv.), Pd[PPh_3_]_4_ (15 mg, 0.01 mmol, 0.05 equiv.), and K_2_CO_3_ (0.9 mL, 2.0 M aqueous solution, 1.72 mmol, 6.6 equiv.) in THF (2 mL), according to the general procedure for Suzuki couplings (conditions B), as described above. Purification by flash column chromatography (silica gel, 20% EtOAc in hexanes) afforded **4** (39 mg, 92%) as a pale yellow oil: R*_f_* = 0.48 (silica gel, 50% EtOAc in hexanes); ^1^H NMR (400 MHz, MeOD) δ 6.60 (d, *J* = 8.6 Hz, 1 H), 6.56 (d, *J* = 3.0 Hz, 1 H), 6.49 (ddd, *J* = 8.6, 3.0, 0.4 Hz, 1 H), 6.21–6.14 (m, 1 H), 1.88 (d, *J* = 1.4 Hz, 3 H), 1.76 (d, *J* = 1.1 Hz, 3 H); ^13^C NMR (100 MHz, MeOD) δ 150.7, 148.7, 136.3, 127.6, 121.9, 117.7, 116.8, 114.9, 26.5, 19.6; HRMS (H-ESI) *m*/*z*: [M + H]^+^ calculated for C_10_H_13_O_2_ 165.0916; found 165.0938.

2-(3-Methylbut-2-en-2-yl)benzene-1,4-diol (**5**). Compound **5** was prepared from bromohydroquinone **20** (50 mg, 0.26 mmol, 1.0 equiv.) by treatment with 3-Methyl-2-buten-2-ylboronic acid pinacol ester (0.06 mL, 0.29 mmol, 1.1 equiv.), Pd[PPh_3_]_4_ (15 mg, 0.01 mmol, 0.05 equiv.), and K_2_CO_3_ (0.9 mL, 2.0 M aqueous solution, 1.72 mmol, 6.6 equiv.) in THF (2 mL), according to the general procedure for Suzuki coupling (conditions B). Purification by flash column chromatography (silica gel, 5% EtOAc in hexanes → 15% EtOAc in hexanes) gave **5** (15 mg, 32%) as a pale yellow oil: R*_f_* = 0.40 (silica gel, 50% EtOAc in hexanes); ^1^H NMR (400 MHz, CD_3_OD) δ 6.61 (d, *J* = 8.7 Hz, 1 H), 6.50 (dd, *J* = 8.6, 3.0 Hz, 1 H), 6.37 (d, *J* = 2.9 Hz, 1 H), 1.86 (dd, *J* = 1.4, 1.0 Hz, 3 H), 1.79 (d, *J* = 0.5 Hz, 3 H), 1.50 (dd, *J* = 2.7, 1.3 Hz, 3 H); ^13^C NMR (100 MHz, CD_3_OD) δ 149.7, 146.1, 132.4, 128.1, 126.2, 115.7, 115.6, 113.4, 20.7, 18.7, 18.6; HRMS (H-ESI) *m*/*z*: [M + H]^+^ calculated for C_11_H_15_O_2_ 179.1072; found 179.1065.

(*E*)-2-(3,3-Dimethylbut-1-en-1-yl)benzene-1,4-diol (**6**). Compound **6** was prepared from bromohydroquinone **12** (100 mg, 0.53 mmol, 1.0 equiv.) by treatment with 2-*t*-Butyl-*E*-vinylboronic acid pinacol ester (0.14 mL, 0.58 mmol, 1.1 equiv.), Pd[PPh_3_]_4_ (31 mg, 0.03 mmol, 0.05 equiv.), and K_2_CO_3_ (1.75 mL, 2.0 M aqueous solution, 3.49 mmol, 6.6 equiv.) in THF (4 mL), according to the general procedure for Suzuki coupling (conditions C). Purification by flash column chromatography (silica gel, 15% EtOAc in hexanes) afforded **6** (80 mg, 79%) as a pale yellow oil: R*_f_* = 0.33 (silica gel, 50% EtOAc in hexanes); ^1^H NMR (400 MHz, CD_3_OD) δ 6.81 (t, *J* = 3.0 Hz, 1 H), 6.58 (dd, *J* = 12.4, 3.9 Hz, 2 H), 6.47 (dd, *J* = 8.6, 2.9 Hz, 1 H), 6.14 (d, *J* = 16.3 Hz, 1 H), 1.11 (s, 9 H); ^13^C NMR (100 MHz, CD_3_OD) δ 151.2, 148.6, 141.9, 127.2, 120.8, 117.3, 115.5, 112.9, 34.3, 30.1; HRMS (H-ESI) *m*/*z*: [M + H]^+^ calculated for C_12_H_17_O_2_ 193.1229; found 193.1238.

(*E*)-2-(3-Methoxyprop-1-en-1-yl)benzene-1,4-diol (**7**). Compound **7** was prepared from bromohydroquinone **20** (50 mg, 0.26 mmol, 1.0 equiv.) by treatment with *trans*-3-Methoxy-1-propenylboronic acid pinacol ester (0.06 mL, 0.29 mmol, 1.1 equiv.), Pd[PPh_3_]_4_ (15 mg, 0.01 mmol, 0.05 equiv.), and K_2_CO_3_ (0.90 mL, 2.0 M aqueous solution, 1.72 mmol, 6.6 equiv.) in THF (2 mL), according to the general procedure for Suzuki coupling (conditions C). Purification by flash column chromatography (silica gel, 25% EtOAc in hexanes) gave **7** (26 mg, 56%) as a pale yellow oil: R*_f_* = 0.30 (silica gel, 50% EtOAc in hexanes); ^1^H NMR (400 MHz, CD_3_OD) δ 6.88–6.82 (m, 2 H), 6.62 (d, *J* = 8.5 Hz, 1 H), 6.54 (dd, *J* = 8.6, 2.9 Hz, 1 H), 6.22 (dt, *J* = 16.0, 6.3 Hz, 1 H), 4.07 (dd, *J* = 6.3, 1.4 Hz, 2 H), 3.36 (s, 3 H); ^13^C NMR (100 MHz, CD_3_OD) δ 151.2, 149.2, 129.4, 126.1, 125.6, 117.5, 116.7, 113.6, 74.7, 57.9; HRMS (H-ESI) *m*/*z*: [M + H]^+^ calculated for C_10_H_13_O_3_ 181.0865; found 181.0884.

2-Methyl-6-(2-methylprop-1-en-1-yl)benzene-1,4-diol (**8**). Compound **8** was prepared from bromo derivative **22** (58 mg, 0.29 mmol, 1.0 equiv.) by treatment with 2-methyl-1-propenylboronic acid pinacol ester (0.12 mL, 0.58 mmol, 2.0 equiv.), Pd[PPh_3_]_4_ (66 mg, 0.06 mmol, 0.2 equiv.), and K_2_CO_3_ (142 mg, 1.03 mmol, 3.6 equiv.) in 1,4-dioxane (1.5 mL), according to the general procedure for Suzuki coupling (conditions A). Purification by flash column chromatography (silica gel, 20% EtOAc in hexanes) afforded **8** (29 mg, 58%) as a yellow oil: R*_f_* = 0.50 (silica gel, 30% EtOAc in hexanes); ^1^H NMR (400 MHz, CD_3_OD) δ 6.44 (dd, *J* = 3.0, 0.5 Hz, 1 H), 6.38–6.36 (m, 1 H), 6.17 (s, 1 H), 2.14 (s, 3 H), 1.89 (d, *J* = 1.2 Hz, 3 H), 1.73 (d, *J* = 1.0 Hz, 3 H); ^13^C NMR (100 MHz, CD_3_OD) δ 149.1, 145.0, 135.9, 126.4, 125.7, 120.5, 115.3, 113.6, 24.9, 18.2, 15.4; HRMS (H-ESI) *m*/*z*: [M + H]^+^ calculated for C_11_H_15_O_2_ 179.1072; found 179.1081.

2-Methyl-6-(3-methylbut-2-en-2-yl)benzene-1,4-diol (**9**). Compound **9** was prepared from bromo derivative **22** (50 mg, 0.24 mmol, 1.0 equiv.) by treatment with 3-methyl-2-buten-2-ylboronic acid pinacol ester (0.06 mL, 0.26 mmol, 1.1 equiv.), Pd[PPh_3_]_4_ (14 mg, 0.01 mmol, 0.05 equiv.) and K_2_CO_3_ (0.8 mL, 2.0 M aqueous solution, 1.72 mmol, 6.6 equiv.) in THF (2 mL), according to the general procedure for Suzuki coupling (conditions B). Purification by flash column chromatography (silica gel, 10% EtOAc in hexanes) gave **9** (16 mg, 35%) as a pale yellow oil: R*_f_* = 0.60 (silica gel, 30% EtOAc in hexanes); ^1^H NMR (400 MHz, CDCl_3_) δ 6.52 (dd, *J* = 3.0, 0.7 Hz, 1 H), 6.33 (dd, *J* = 3.1, 0.5 Hz, 1 H), 2.21 (t, *J* = 0.6 Hz, 3 H), 1.89 (dd, *J* = 1.5, 1.0 Hz, 3 H), 1.85 (d, *J* = 0.7 Hz, 3 H), 1.55 (q, *J* = 1.4 Hz, 3 H); ^13^C NMR (100 MHz, CDCl_3_) δ 148.4, 143.8, 132.1, 130.7, 124.7, 124.6, 116.1, 112.2, 21.7, 20.1, 20.0, 16.3; HRMS (H-ESI) *m*/*z*: [M + H]^+^ calculated for C_12_H_17_O_2_ 193.1229; found 193.1234.

(*E*)-2-(3,3-Dimethylbut-1-en-1-yl)-6-methylbenzene-1,4-diol (**10**). Compound **10** was prepared from bromo derivative **22** (100 mg, 0.53 mmol, 1.0 equiv.), by treatment with 2-*t*-Butyl-*E*-vinylboronic acid pinacol ester (0.14 mL, 0.58 mmol, 1.1 equiv.), Pd[PPh_3_]_4_ (31 mg, 0.03 mmol, 0.05 equiv.), and K_2_CO_3_ (1.75 mL, 2.0 M aqueous solution, 3.50 mmol, 6.6 equiv.) in THF (4 mL), according to the general procedure for Suzuki coupling (conditions B). Purification by flash column chromatography (silica gel, 15% EtOAc in hexanes) gave **10** (40 mg, 79%) as a pale yellow oil: R*_f_* = 0.40 (silica gel, 30% EtOAc in hexanes); ^1^H NMR (400 MHz, CDCl_3_) δ 6.67–6.65 (m, 1 H), 6.52 (dd, *J* = 3.0, 0.6 Hz, 1 H), 6.43 (d, *J* = 16.2 Hz, 1 H), 6.16 (d, *J* = 16.2 Hz, 1 H), 2.20 (t, *J* = 0.6 Hz, 3 H), 1.12 (s, 9 H); ^13^C NMR (100 MHz, CDCl_3_) δ 148.8, 144.9, 144.9, 125.6, 125.2, 118.8, 116.3, 110.9, 33.7, 29.5, 16.1; HRMS (H-ESI) *m*/*z*: [M + H]^+^ calculated for C_13_H_19_O_2_ 207.1385; found 207.1367.

(*E*)-2-(3-Methoxyprop-1-en-1-yl)-6-methylbenzene-1,4-diol (**11**). Compound **10** was prepared from bromo derivative **22** (50 mg, 0.25 mmol, 1.0 equiv.) by treatment with *trans*-3-methoxy-1-propenylboronic acid pinacol ester (0.10 mL, 0.49 mmol, 2.0 equiv.), Pd[PPh_3_]_4_ (57 mg, 0.05 mmol, 0.2 equiv.), and K_2_CO_3_ (122 mg, 0.89 mmol, 3.6 equiv.) in 1,4-dioxane (1.5 mL), according to the general procedure for Suzuki coupling (conditions A). Purification by flash column chromatography (silica gel, 25% EtOAc in hexanes) afforded **11** (24 mg, 51%) as a pale yellow oil: R*_f_* = 0.30 (silica gel, 50% EtOAc in hexanes); ^1^H NMR (400 MHz, CD_3_OD) δ 6.97–6.90 (m, 1 H), 6.71 (d, *J* = 3.0 Hz, 1 H), 6.50 (dd, *J* = 3.0, 0.7 Hz, 1 H), 6.16 (dt, *J* = 15.9, 6.3 Hz, 1 H), 4.08 (dd, *J* = 6.3, 1.4 Hz, 2 H), 3.37 (s, 3 H), 2.15 (s, 3 H); ^13^C NMR (100 MHz, CD_3_OD) δ 150.1, 145.2, 128.2, 127.1, 125.6, 124.9, 116.9, 109.3, 73.2, 56.6, 15.5; HRMS (H-ESI) *m*/*z*: [M + H]^+^ calculated for C_11_H_15_O_3_ 195.1021; found 195.1023.

#### 3.3.5. Synthesis of Compounds **23**, **24**, and 2,5-Disubstituted Hydroquinones **12** and **15**

2-Bromo-5-methylcyclohexa-2,5-diene-1,4-dione (**23**). To a solution of toluquinol (**1**) (2.5 g, 20.14 mmol, 1.0 equiv.) in acetone (15 mL) was added K_2_CO_3_ (14 g, 100.70 mmol, 5.0 equiv.) and Me_2_SO_4_ (5.7 mL, 60.41 mmol, 3.0 equiv.) and the reaction mixture was stirred for 3 h. After this time, the reaction mixture was diluted with water and the aqueous phase was extracted with Et_2_O. The organic phase was washed with brine, dried over MgSO_4_, filtered, and the solvent removed under reduced pressure to obtain the corresponding dimethoxy derivative (~20 mmol), which was used in the next steps without purification. To a solution of the dimethoxy derivative obtained above (~20 mmol) and NaOAc (3.3 g, 40.28 mmol, 2.0 equiv.) in AcOH (20 mL) was added bromine (1.2 mL, 2.15 mmol, 1.1 equiv.) over 25 min and, after the addition, the reaction mixture was stirred for 1 h. Then, the reaction mixture was quenched by a slow addition of a saturated aqueous NaHCO_3_ solution at 0 °C. The aqueous phase was then extracted with EtOAc and the organic phase washed with brine, dried over MgSO_4_, filtered, and the solvent removed under reduced pressure to obtain the corresponding bromo derivative (~20 mmol), which was used in the next step without purification. The bromo derivative obtained above (~20 mmol) was dissolved in CH_3_CN (35 mL). Then, CAN (28 g, 50.34 mmol, 2.5 equiv.) and H_2_O (20 mL) were added and the reaction mixture was stirred for 1 h at 25 °C. After this time, the reaction mixture was diluted with water and the aqueous phase was extracted with Et_2_O twice. The combined organic phases were washed with brine, dried over MgSO_4_, filtered, and the solvent removed under reduced pressure. The residue was purified by flash column chromatography (silica gel, 1% EtOAc in hexanes) to obtain compound **23** (1.5 g, 37% over 3 steps) as an orange solid [13]: R*_f_* = 0.45 (silica gel, 20% EtOAc in hexanes); ^1^H NMR (400 MHz, CDCl_3_) δ 7.29 (s, 1 H), 7.26 (s, 2 H), 2.08 (d, *J* = 1.6 Hz, 3 H); ^13^C NMR (100 MHz, CDCl_3_) δ 185.1, 179.5, 146.5, 138.1, 137.5, 132.6, 15.7.

2-Bromo-5-methylbenzene-1,4-diol (**24**). Quinone **23** (264 mg, 1.31 mmol, 1.0 equiv.) was dissolved in a mixture of EtOH/H_2_O (3/0.5 mL) and heated to 50 °C. Then, Na_2_S_2_O_4_ (264 mg, 1.57 mmol, 1.2 equiv.) was added and the reaction mixture was stirred at 50 °C for 1.5 h. After this time, the reaction mixture was cooled to 25 °C and the EtOH was removed under reduced pressure. The resulting residue was diluted with water and extracted with EtOAc twice and the combined organic phases washed with brine, dried over MgSO_4_, filtered, and the solvent removed under reduced pressure to obtain a yellow solid. This solid was washed with cold CH_2_Cl_2_ to afford pure compound **24** (118 mg, 44%) as a pale yellow solid that did not require further purification: R*_f_* = 0.52 (silica gel, 50% EtOAc in hexanes); ^1^H NMR (400 MHz, CD_3_OD) δ 6.83 (s, 1 H), 6.64 (d, *J* = 0.7 Hz, 1 H), 2.08 (s, 3 H); ^13^C NMR (100 MHz, CD_3_OD) δ 148.7, 146.4, 124.9, 117.9, 117.8, 105.6, 14.6; HRMS (H-ESI) *m*/*z*: [M + H]^+^ calculated for C_7_H_8_BrO_2_ 202.9708; found 202.9715.

(*E*)-2-(3-Methoxyprop-1-en-1-yl)-5-methylbenzene-1,4-diol (**12**). Compound **12** was prepared from bromo derivative **24** (113 mg, 0.56 mmol, 1.0 equiv.) by treatment with *trans*-3-Methoxy-1-propenylboronic acid pinacol ester (0.24 mL, 1.11 mmol, 2.0 equiv.), Pd[PPh_3_]_4_ (129 mg, 0.11 mmol, 0.2 equiv.), and K_2_CO_3_ (277 mg, 2.00 mmol, 3.6 equiv.) in 1,4-dioxane (3 mL), according to the general procedure for Suzuki coupling (conditions A). Purification by flash column chromatography (silica gel, 5% EtOAc in hexanes → 20% EtOAc in hexanes) gave compound **12** (54 mg, 50%) as a pale yellow oil: R*_f_* = 0.50 (silica gel, 50% EtOAc in hexanes); ^1^H NMR (400 MHz, CD_3_OD) δ 6.85–6.78 (m, 2 H), 6.52 (d, *J* = 0.6 Hz, 1 H), 6.15 (dt, *J* = 15.9, 6.5 Hz, 1 H), 4.06 (dd, *J* = 6.4, 1.3 Hz, 2 H), 3.35 (s, 3 H), 2.11 (s, 3 H); ^13^C NMR (100 MHz, CD_3_OD) δ 147.9, 147.5, 128.2, 125.4, 123.4, 121.4, 117.6, 111.7, 73.5, 56.5, 14.8; HRMS (H-ESI) *m*/*z*: [M + H]^+^ calculated for C_11_H_15_O_3_ 195.1021; found 195.1017.

Thymoquinol **15**. To a solution of thymoquinone (**16**) (100 mg, 0.61 mmol, 1.0 equiv.) in MeOH (2 mL) was added a solution of Na_2_S_2_O_4_ (225 mg, 1.29 mmol, 2.1 equiv.) in H_2_O (2 mL). The reaction mixture was stirred for 2 h at 25 °C and, after this time, the organic solvent was removed under reduced pressure. Then, the crude mixture was diluted with water and the aqueous phase was extracted with EtOAc. The organic phase was washed with brine, dried over MgSO_4_, filtered, and the solvent removed under reduced pressure. The residue was purified by flash column chromatography (silica gel, 20% EtOAc in hexanes) to obtain **15** (50 mg, 49%) as a pale brown solid [14]: R*_f_* = 0.50 (silica gel, 30% EtOAc in hexanes); ^1^H NMR (400 MHz, CD_3_OD) δ 6.56 (s, 1 H), 6.48 (s, 1 H), 3.17 (dt, *J* = 13.8, 6.9 Hz, 1 H), 1.17 (s, 3 H), 1.15 (s, 3 H); ^13^C NMR (100 MHz, CD_3_OD) δ 147.8, 146.6, 132.8, 121.6, 116.9, 112.1, 26.3, 21.8, 14.5; HRMS (H-ESI) *m*/*z*: [M + H]^+^ calculated for C_10_H_15_O_2_ 167.1072; found 167.1058.

### 3.4. Cell Growth Assay

The 3-(4,5-dimethylthiazol-2-yl)-2,5-diphenyltetrazolium bromide or MTT dye reduction assay in 96-well microplates was used [32]. This assay is dependent on the reduction of MTT by mitochondrial dehydrogenases of a viable cell to a blue formazan product, which can be measured spectrophotometrically. 3 × 10^3^ HL-60, and 2 × 10^3^ HT-1080, HT-29, MDA-MB-231, and U87MG cells in a total volume of 100 μL of their respective growth medium were incubated with serial dilutions 1:1 of the tested compounds. After three days of incubation (37 °C and 5% CO_2_ in a humid atmosphere), 10 μL of MTT (5 mg/mL in phosphate-buffered saline) were added to each well, and the plate was incubated for a further 4 h at 37 °C. The resulting formazan was dissolved in 150 μL of 0.04 N HCl/2-propanol and read at 550 nm. IC_50_ values were calculated from semi-logarithmic dose‒response plots as those concentrations of the compound yielding 50% cell survival, taking the values obtained for the control to be 100%. IC_50_ results are expressed as means ± S.D. of at least three independent experiments.

## 4. Conclusions and Future Perspectives

Bioactive marine compounds can serve as the basis for the synthesis of derivatives with improved pharmacological properties. The synthesis of a set of analogues of the natural product toluquinol has been described and their in vitro cytotoxicity against a panel of cancer cell lines was compared. Our results indicate that the cytotoxic activity of this family of compounds could rely on the hydroquinone part of the molecule, probably due to its capability to undergo redox cycling. Although the hydroquinones seemed to be more cytotoxic than their quinone counterparts, the differences between them were not very significant. Substituents in the ring may modulate the interaction of the molecule with their targets, changing either the activity or the selectivity. Tolerance for biological activity was much more restricted for the methyl group of the molecule, whose replacement by other groups resulted in a significant loss of cytotoxic activity. An increased selectivity was obtained when prenyl groups were used as substituents. The inclusion of a second substituent group in the toluquinol molecule was well tolerated and originated a series of derivatives with cytotoxic activities that were similar to or even higher than that of the natural product. These findings provide guidance for the design of new toluquinol analogues with potentially better pharmacological properties. Two of the compounds studied here (toluquinol and thymoquinone) have been reported to inhibit angiogenesis in vitro and in vivo. Further optimization and development of the new compounds and biochemical studies to determine the mechanism of the biological actions of this family of compounds, with an emphasis on their putative antiangiogenic activity and their undesired toxic effects, are currently in progress.

## Figures and Tables

**Table 1:** In vitro antitumor activities of toluquinol and analogues (IC_50_, μM) [a].

Structure	Compound	Tumor Cell Line				
		MDA-MB-231 ^[b]^	HL-60 ^[c]^	U87-MG ^[d]^	HT-1080 ^[e]^	HT-29 ^[f]^
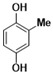	**Toluquinol (1)**	2.3 ± 0.8	1.7 ± 0.5	5.6 ± 1.5	1.4 ± 0.6	4.1 ± 0.4
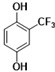	**Compound 2**	14.5 ± 1.8	13.9 ± 1.1	31.7 ± 5.6	41.2 ± 11.4	29.7 ± 7.1
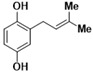	**Compound 3**	>100	50.0 ± 3.4	>100	>100	>100
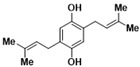	**Compound 18**	>100	7.0 ± 1.2	>100	>100	>100
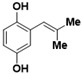	**Compound 4**	4.1 ± 1.1	3.0 ± 0.7	10.4 ± 3.6	3.7 ± 1.8	7.9 ± 0.1
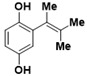	**Compound 5**	3.9 ± 1.5	5.3 ± 2.0	29.7 ± 4.9	8.4 ± 1.4	15.5 ± 4.5
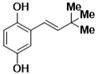	**Compound 6**	6.7 ± 1.8	6.3 ± 1.3	15.9 ± 4.4	6.1 ± 2.5	12.3 ± 2.5
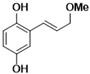	**Compound 7**	4.2 ± 1.8	6.2 ± 1.7	8.8 ± 0.8	3.2 ± 1.9	9.8 ± 3.2
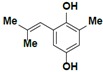	**Compound 8**	2.2 ± 0.3	3.1 ± 1.7	10.0 ± 2.1	3.3 ± 0.1	8.1 ± 0.6
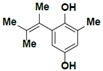	**Compound 9**	2.6 ± 1.3	3.6 ± 1.5	15.8 ± 2.3	5.5 ± 1.9	14.2 ± 4.3
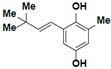	**Compound 10**	3.2 ± 1.2	4.7 ± 0.8	16.0 ± 1.3	5.0 ± 1.5	15.6 ± 1.2
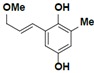	**Compound 11**	2.4 ± 0.3	3.1 ± 1.3	9.6 ± 1.3	2.9 ± 1.0	7.7 ± 1.6
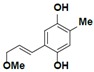	**Compound 12**	1.6 ± 0.5	2.9 ± 1.4	5.2 ± 1.2	1.1 ± 0.2	7.9 ± 2.0
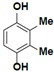	**Compound 13**	2.0 ± 0.3	3.2 ± 1.6	5.1 ± 0.4	1.6 ± 0.3	6.5 ± 2.6
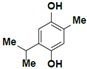	**Thymoquinol (15)**	1.0 ± 0.1	1.6 ± 0.5	10.2 ± 2.0	2.0 ± 1.1	8.2 ± 1.4
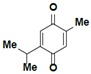	**Thymoquinone (16)**	1.0 ± 0.1	2.0 ± 0.9	15.1 ± 3.2	4.0 ± 0.9	12.9 ± 1.6
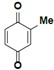	**Toluquinone (14)**	3.4 ± 0.1	5.6 ± 1.9	8.8 ± 0.9	4.9 ± 1.7	9.5 ± 0.5
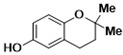	**Compound 19**	>100	82.0 ± 5.9	>100	>100	>100

^[a]^ Half-maximal inhibitory concentration (IC50) values calculated from dose-response curves as the concentration of compound yielding 50% of control cell survival. They are expressed as means + SD of three independent experiments with quadruplicate samples each. ^[b]^ MDA-MB-231: Human breast adenocarcinoma. ^[c]^ HL-60: Human promyelocytic leukemia. ^[d]^ U87-MG: Human glioblastoma. ^[e]^ HT-1080: Human fibrosarcoma. ^[f]^ HT-29: Human colorectal adenocarcinoma.

## Supplementary Materials

The following are available online at https://www.mdpi.com/1660-3397/17/9/492/s1.
