# Supplementary material for: Synthesis and Antitumor Activity Evaluation of Compounds Based on Toluquinol
Source: Mar Drugs. 2019 Aug 23;17(9):492. doi: 10.3390/md17090492 (PMC6780176; doi:10.3390/md17090492)

Supporting Information

Synthesis and Anti-Tumor Activities Evaluation of Compounds Based on Toluquinol

Iván Cheng-Sánchez[1,¥], José A. Torres-Vargas[2,3,¥], Beatriz Martínez-Poveda[2,3], Guillermo A. Guerrero-Vásquez[1], Miguel Ángel Medina[2,3,4], Francisco Sarabia[1,*] and Ana R. Quesada[2,3,4,*]

[1] Department of Organic Chemistry, Faculty of Sciences, University of Málaga, Campus de Teatinos s/n, 29071, Málaga (SPAIN); cheng@uma.es (I. C-S.); guillermo.guerrero@gm.uca.es (G. A. G.-V.); frsarabia@uma.es (F. S.)

[2] Department of Molecular Biology and Biochemistry, Faculty of Sciences, University of Málaga, Campus de Teatinos s/n, 29071, Málaga (SPAIN); joseantoniotorresvargas@gmail.com (J.A.T.-V.); bmpoveda@uma.es (B. M.-P.); medina@uma.es (M. A. M.); quesada@uma.es (A. R. Q.)

[3] IBIMA (Biomedical Research Institute of Málaga), 29071, Málaga (SPAIN)

[4] CIBER of Rare Diseases; Group U741 (CB06/07/0046), 29071 Málaga (SPAIN)

* Correspondence: quesada@uma.es; frsarabia@uma.es; Tel.: +34-952-134-258. Francisco Sarabia and Ana R. Quesada contributed equally to the direction of this work.

¥ Iván Cheng-Sánchez and José A. Torres-Vargas contributed equally to the execution of this work.

Index:	Pages
^{1}H and ^{13}C NMR Spectra	S2-S21
Representative dose-response curves	S22-S30

^1H and ^{13}C NMR Spectra

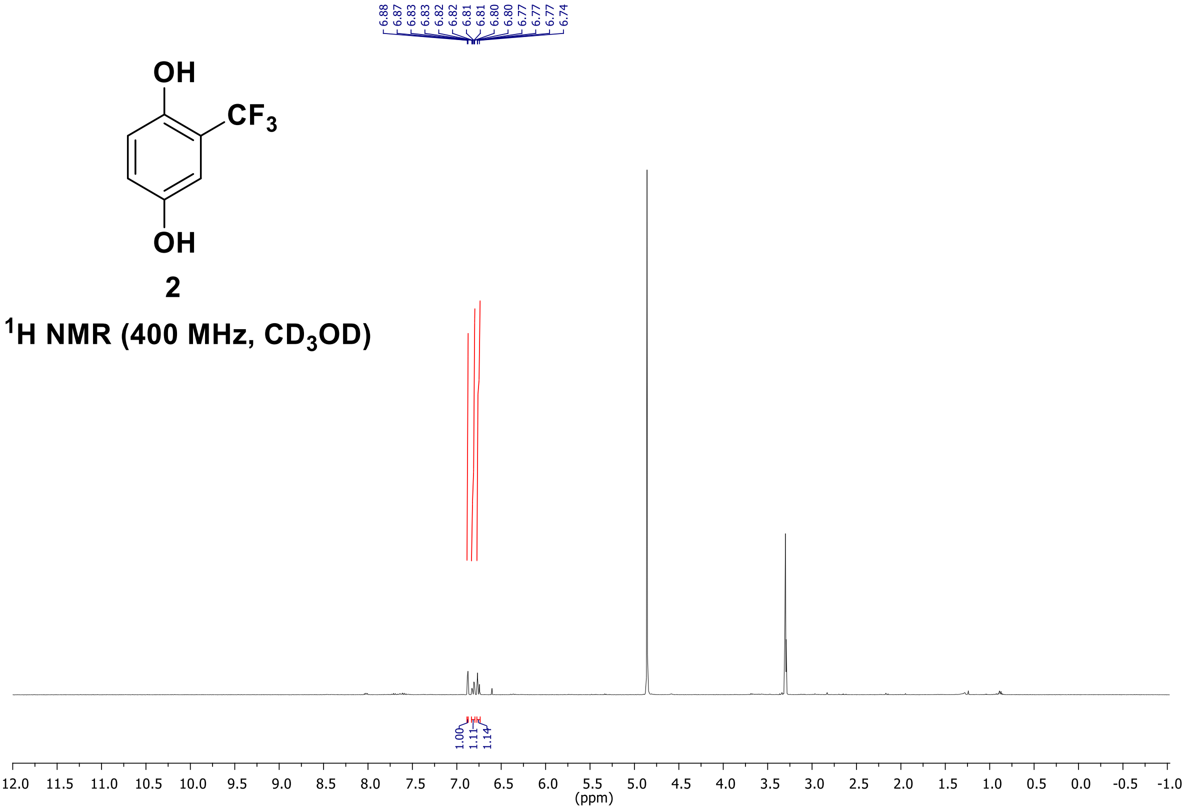

2

^1H NMR (400 MHz, CD_3OD)

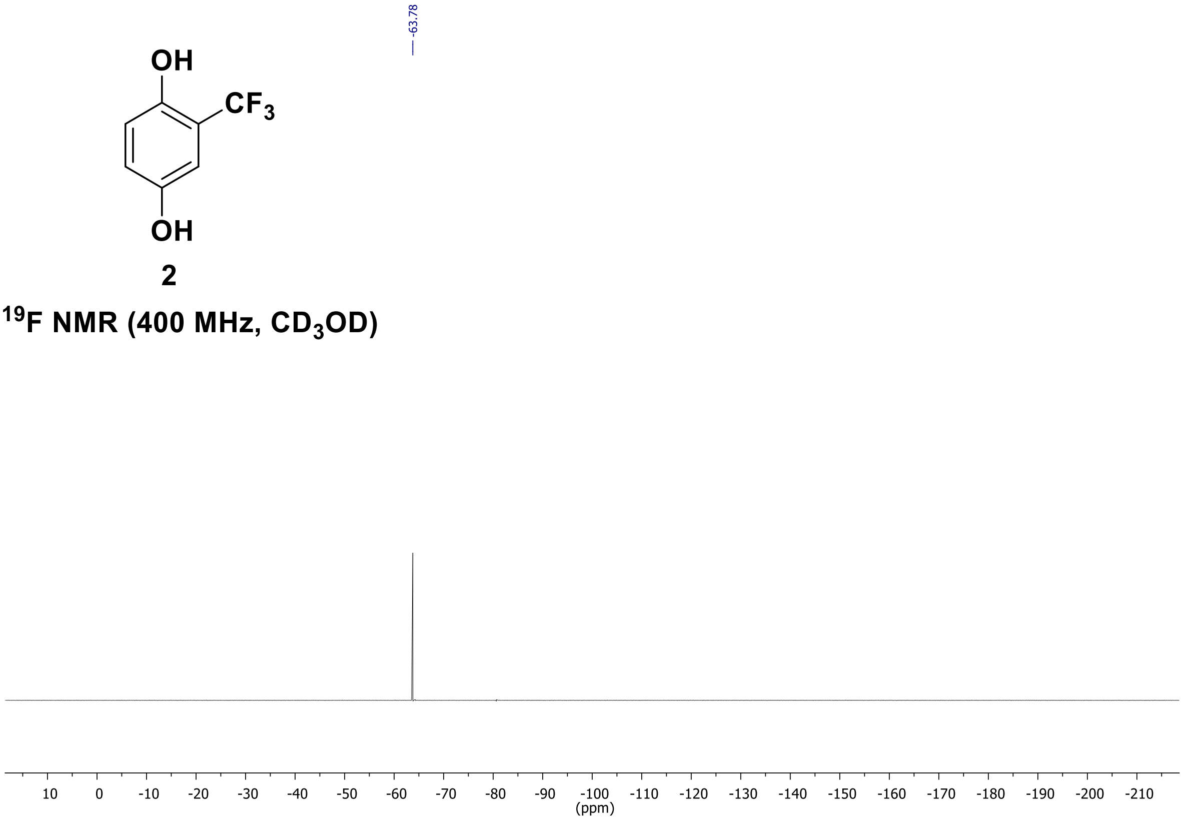

2

^{19}F NMR (400 MHz, CD_3OD)

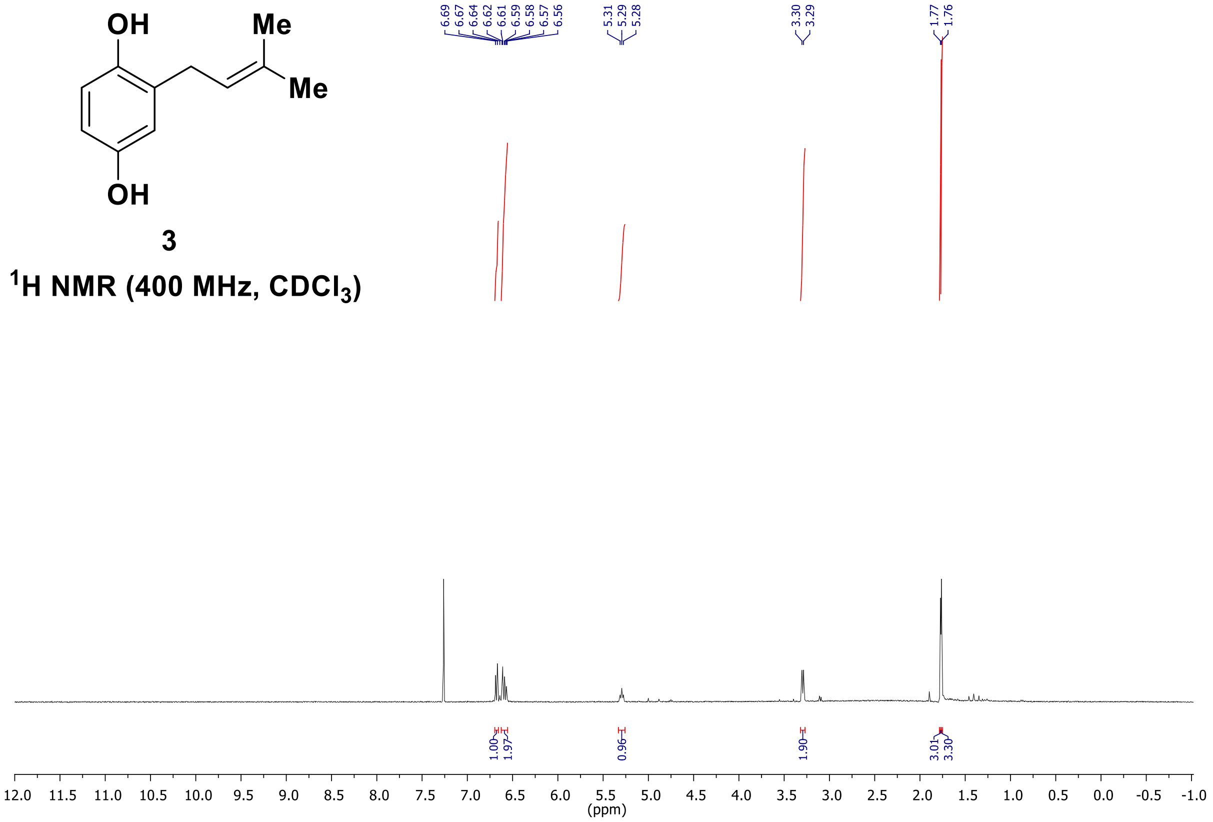

3

^{1}H NMR (400 MHz, $CDCl_3$)

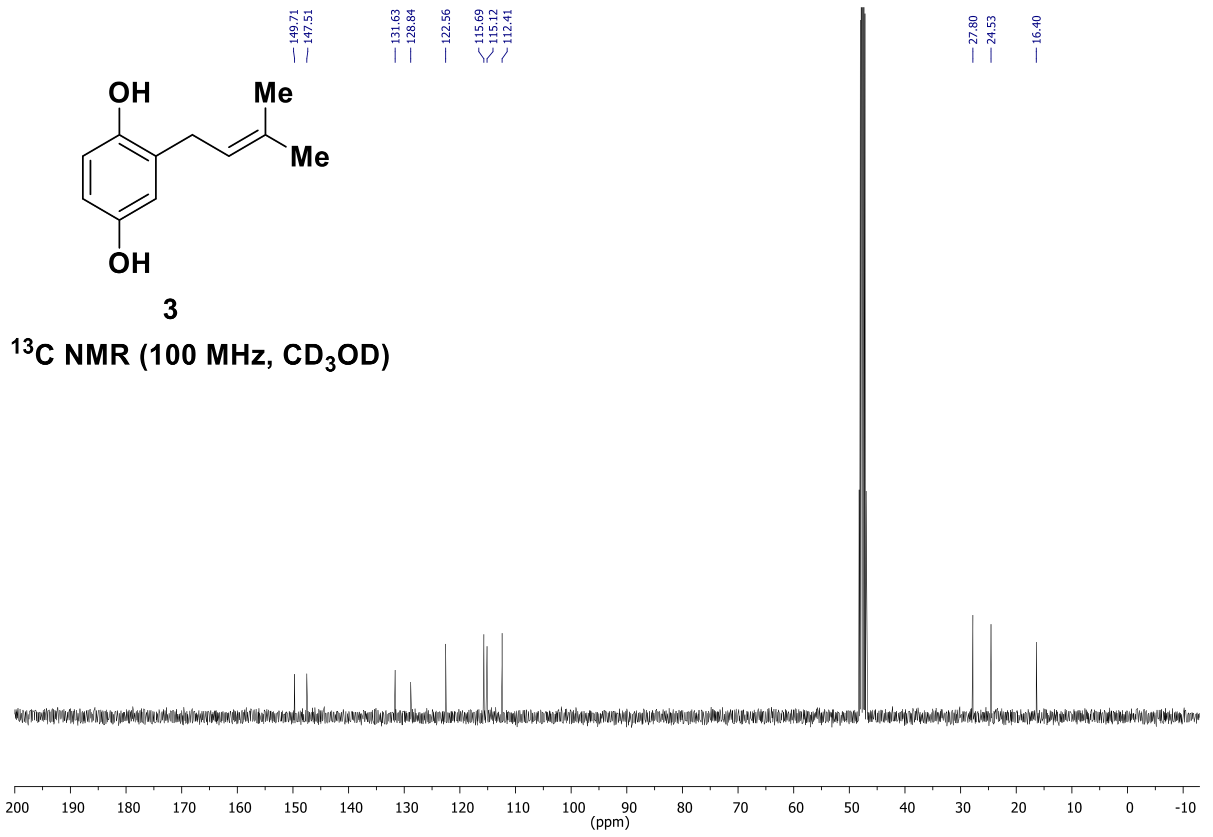

3

^{13}C NMR (100 MHz, CD_3OD)

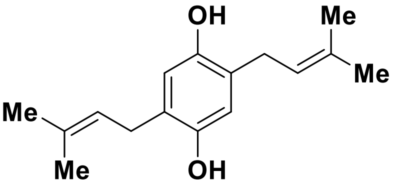

18

^{1}H NMR (400 MHz, $CDCl_3$)

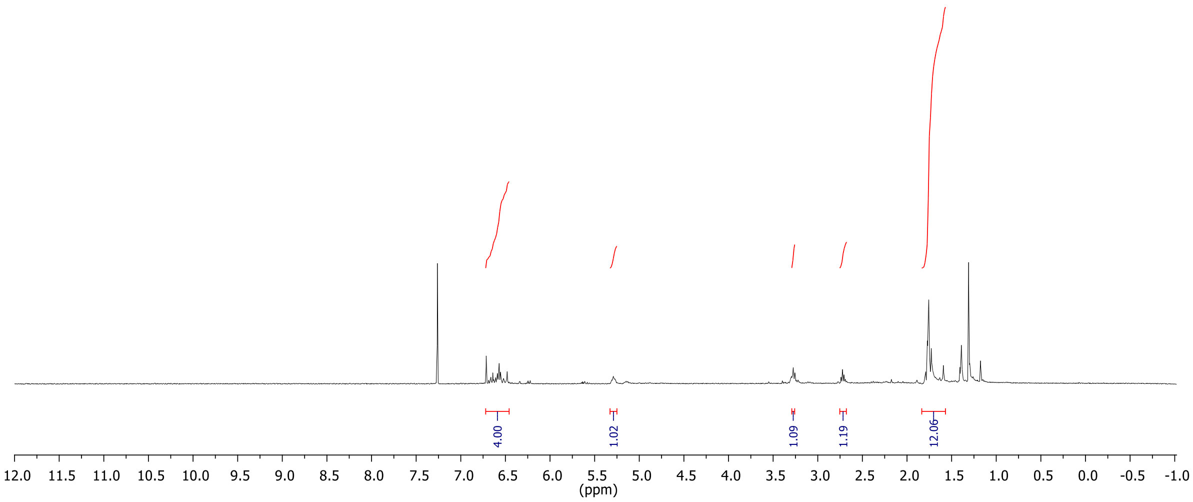

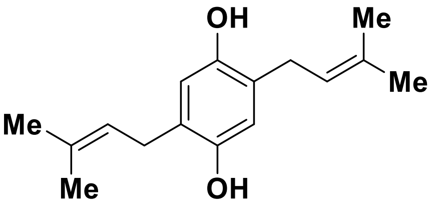

18

^{13}C NMR (100 MHz, CD_3OD)

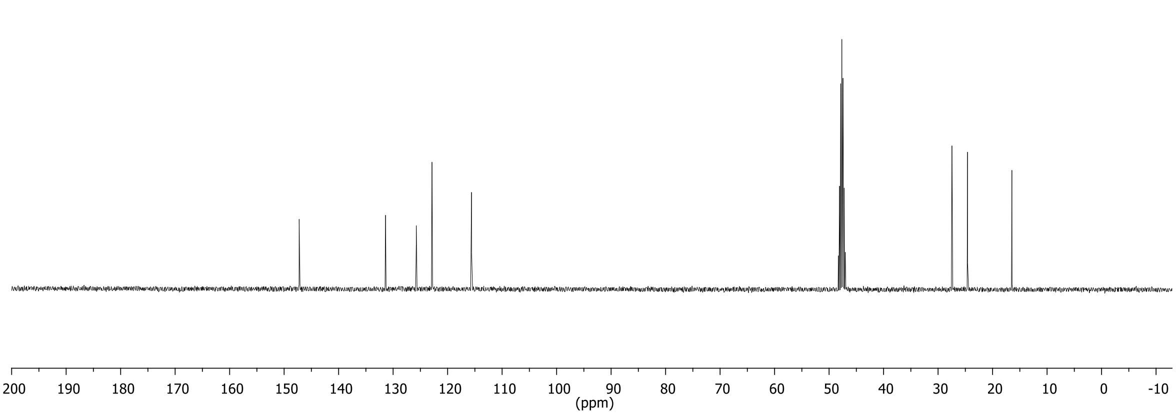

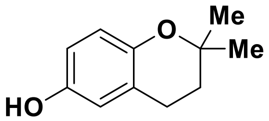

19

^{1}H NMR (400 MHz, $CDCl_3$)

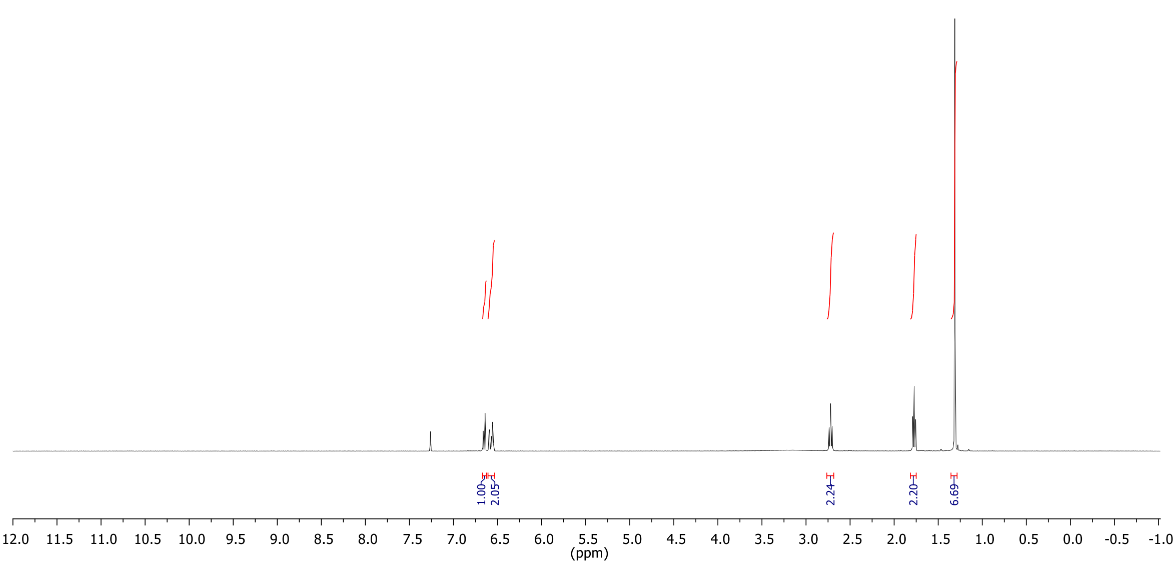

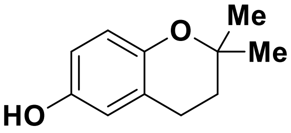

19

^{13}C NMR (100 MHz, CD_3OD)

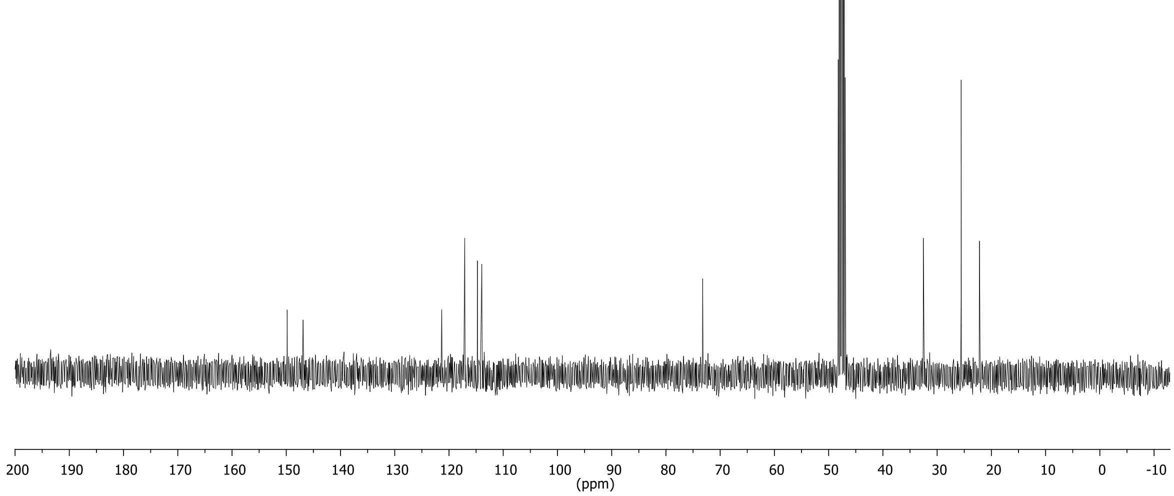

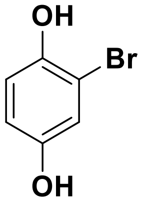

20

^1H NMR (400 MHz, CD_3OD)

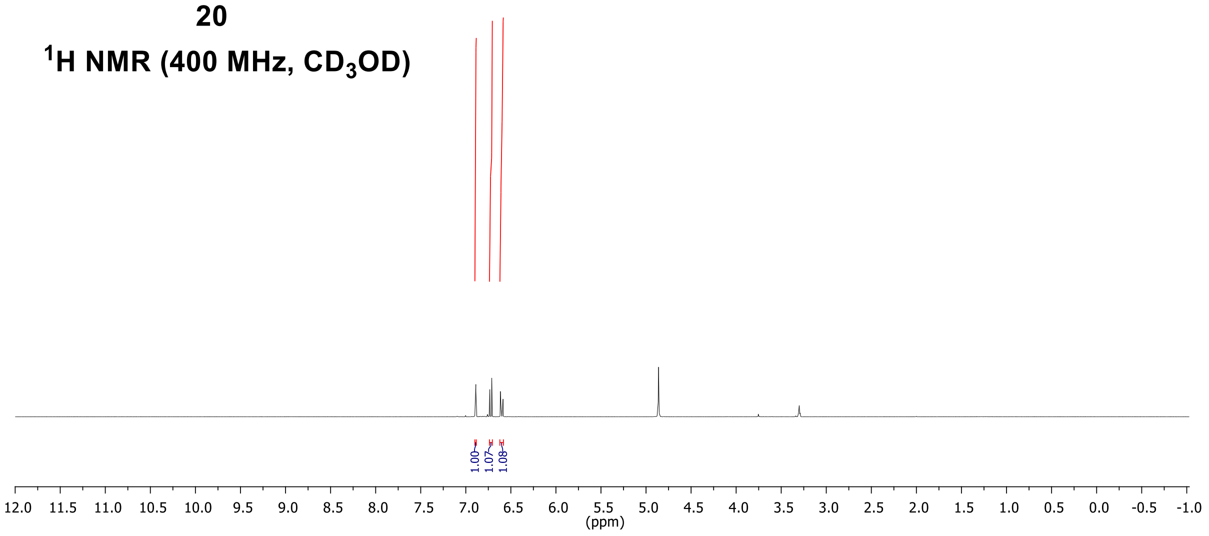

20

^{13}C NMR (100 MHz, CD_3OD)

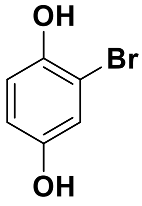

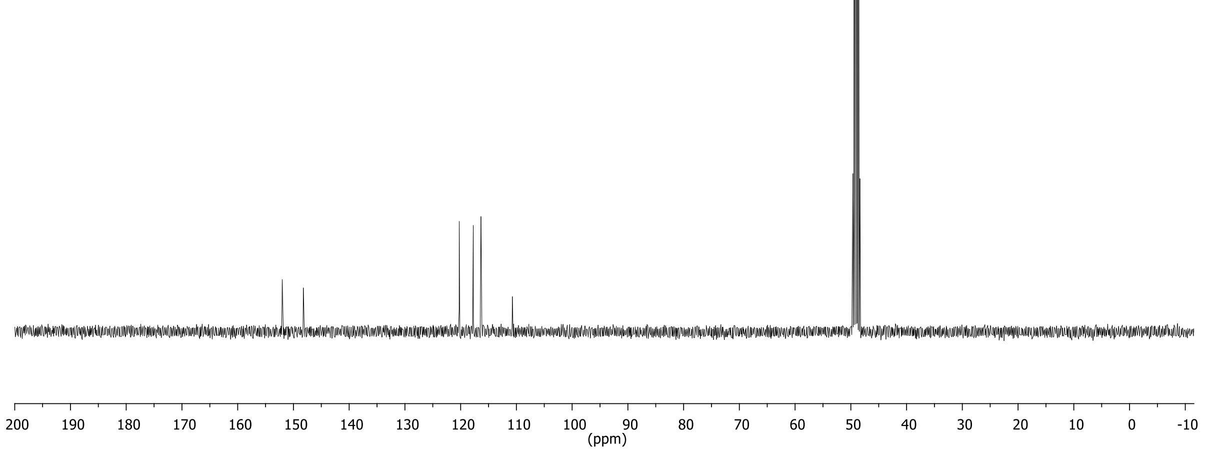

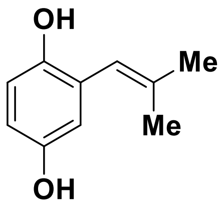

4

^{1}H NMR (400 MHz, CD_3OD)

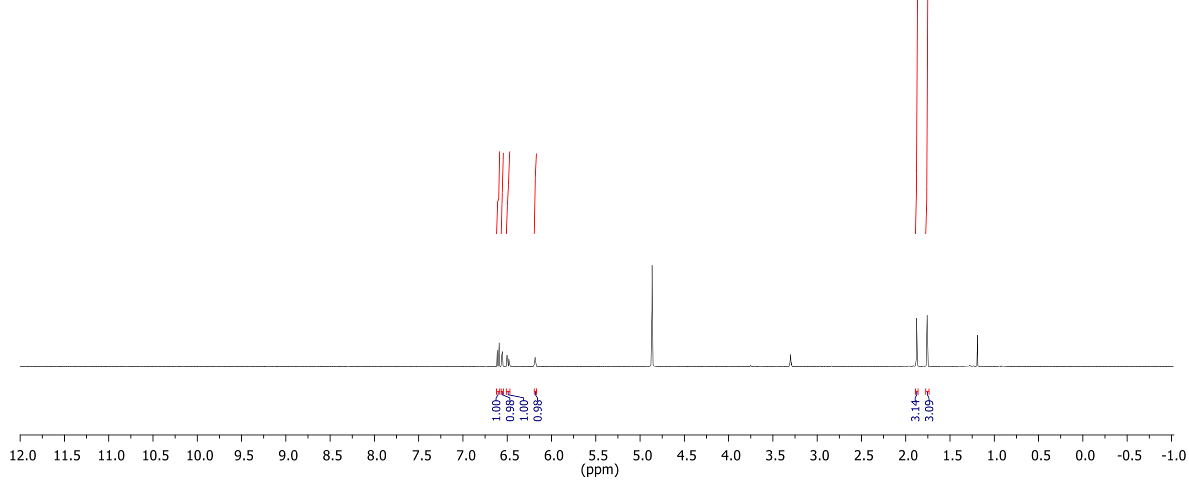

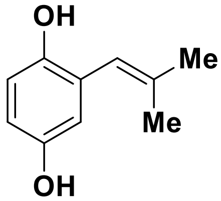

4

^{13}C NMR (100 MHz, CD_3OD)

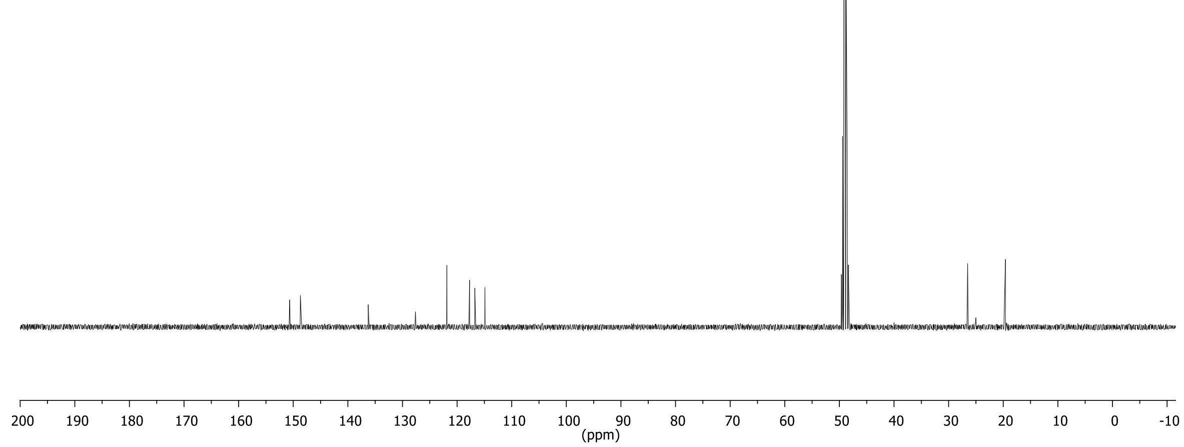

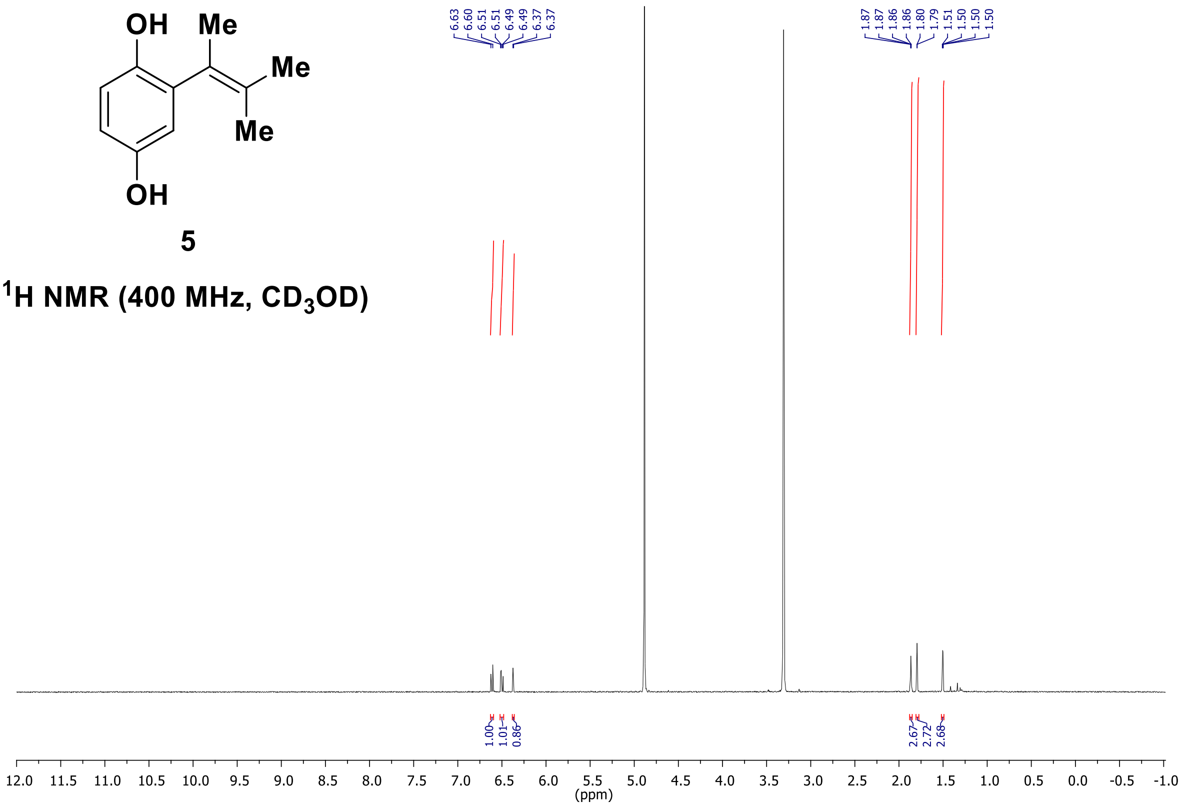

5

^{1}H NMR (400 MHz, CD_3OD)

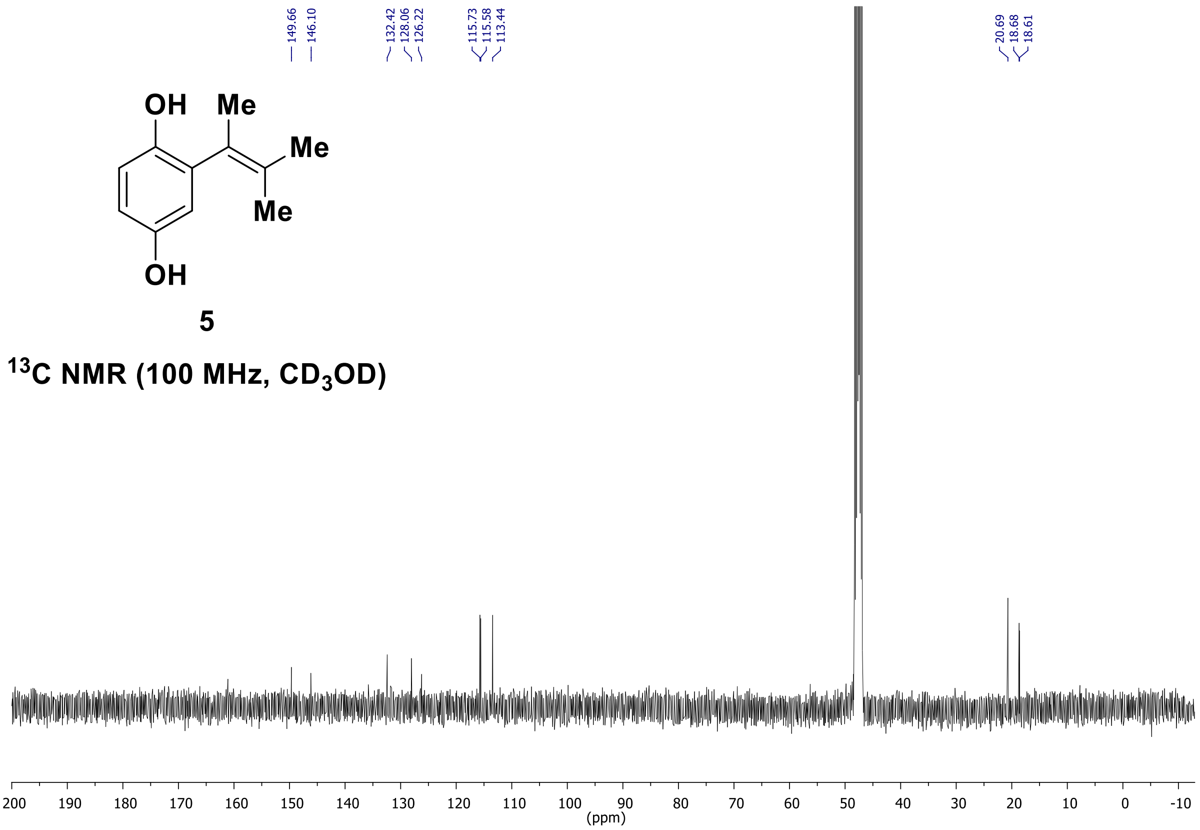

5

^{13}C NMR (100 MHz, CD_3OD)

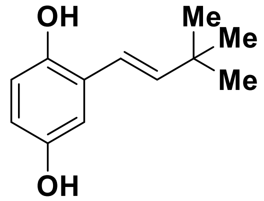

6

^{1}H NMR (400 MHz, CD_3OD)

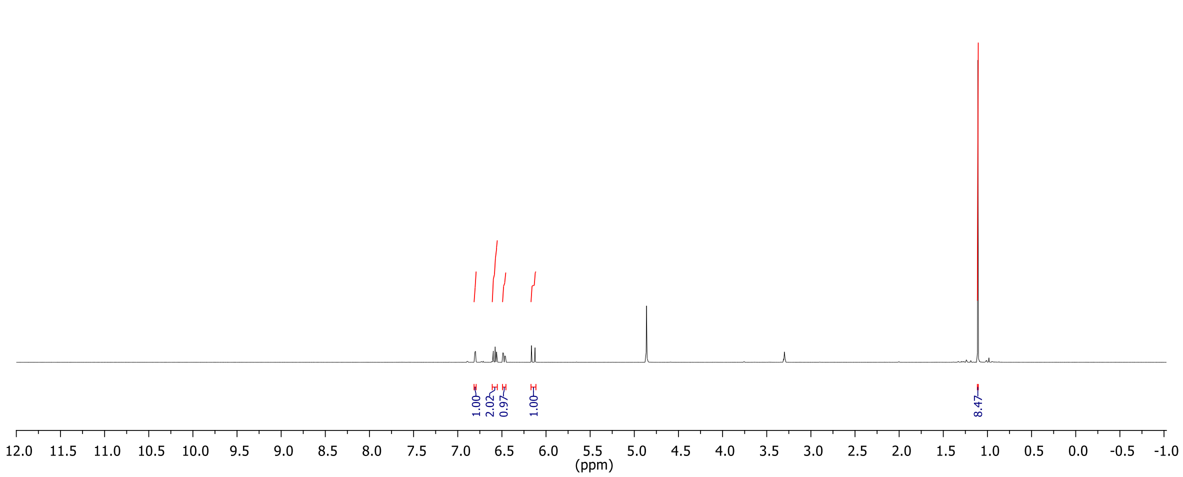

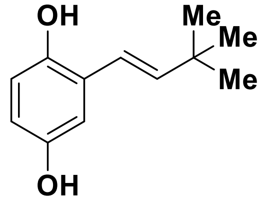

6

^{1}H NMR (400 MHz, CD_3OD)

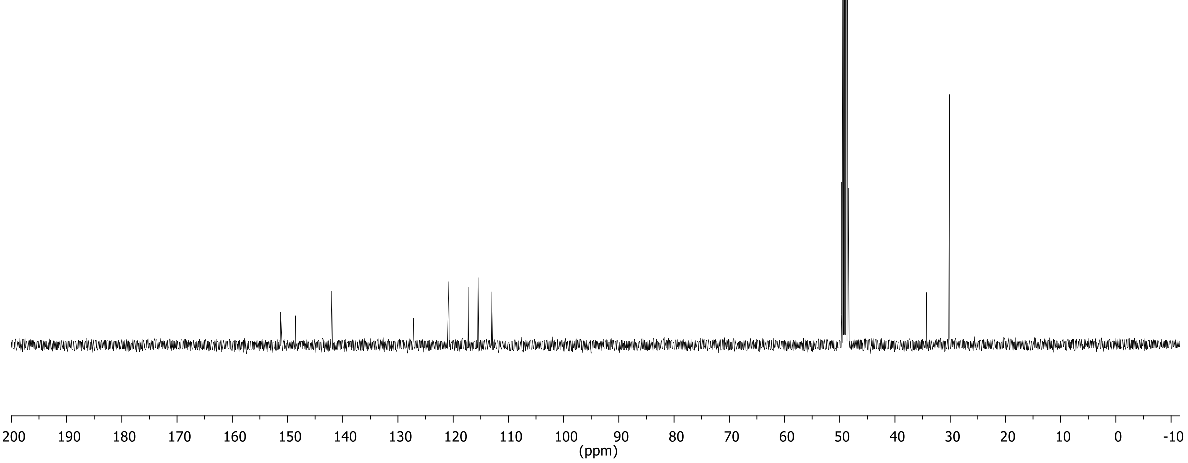

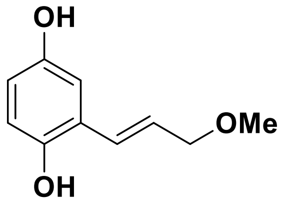

7

^{1}H NMR (400 MHz, CD_3OD)

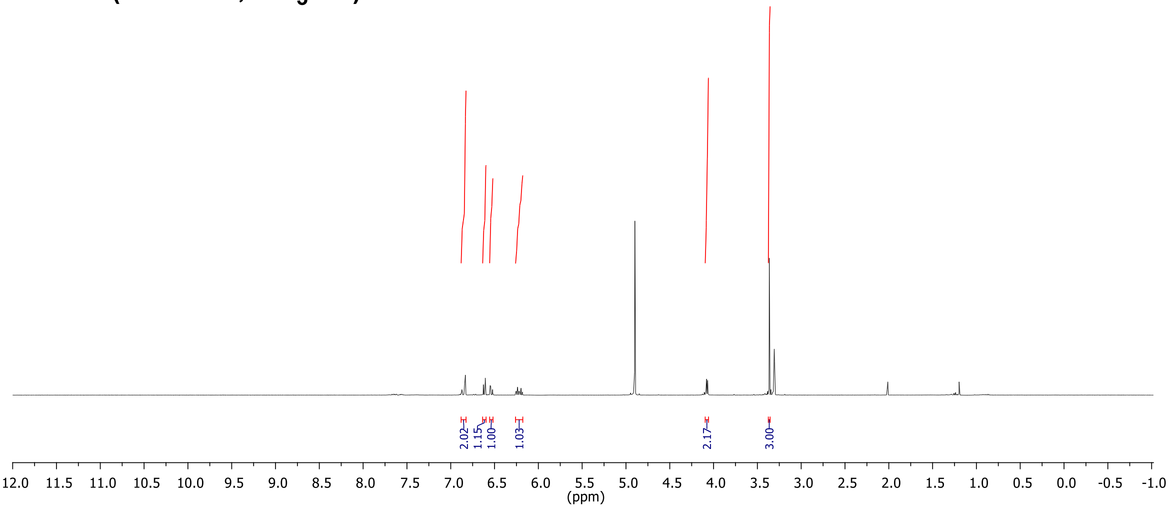

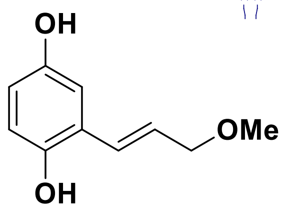

7

^{1}H NMR (400 MHz, CD_3OD)

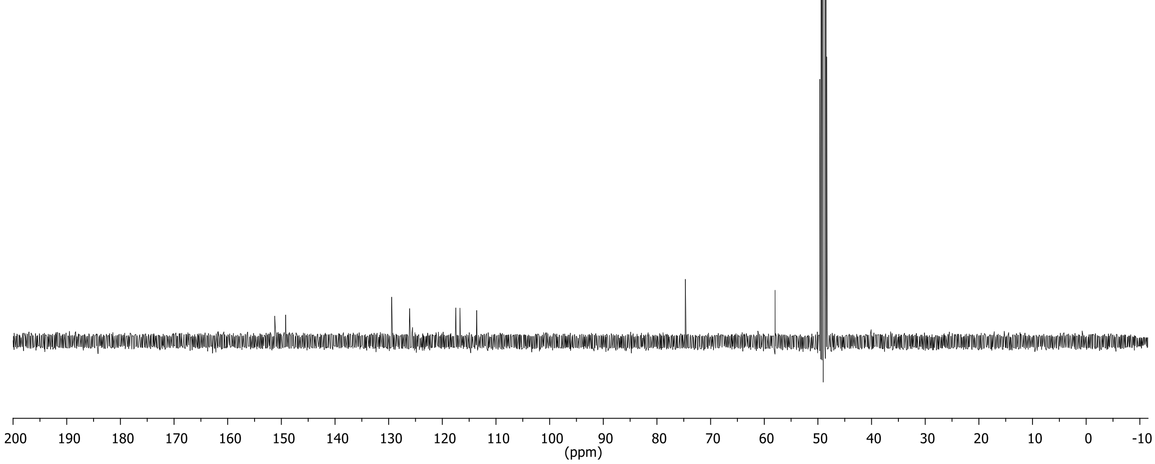

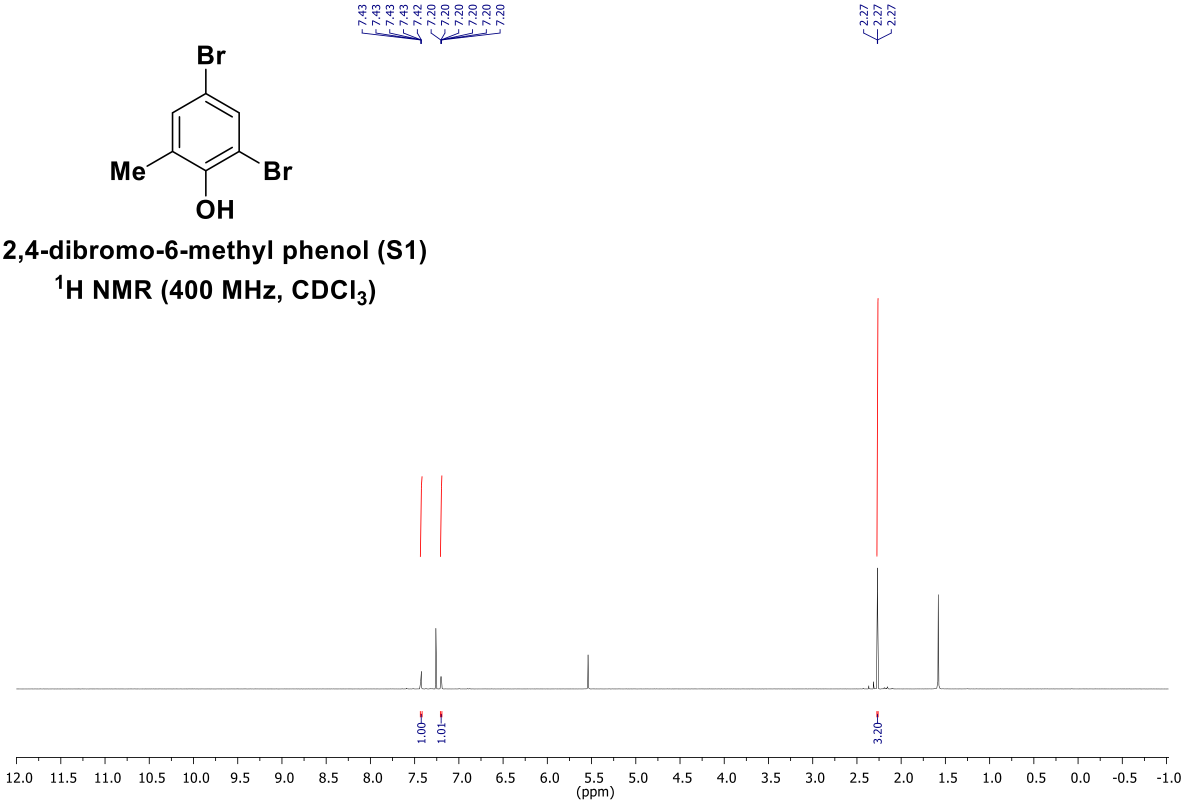

2,4-dibromo-6-methyl phenol (S1)

^{1}H NMR (400 MHz, $CDCl_3$)

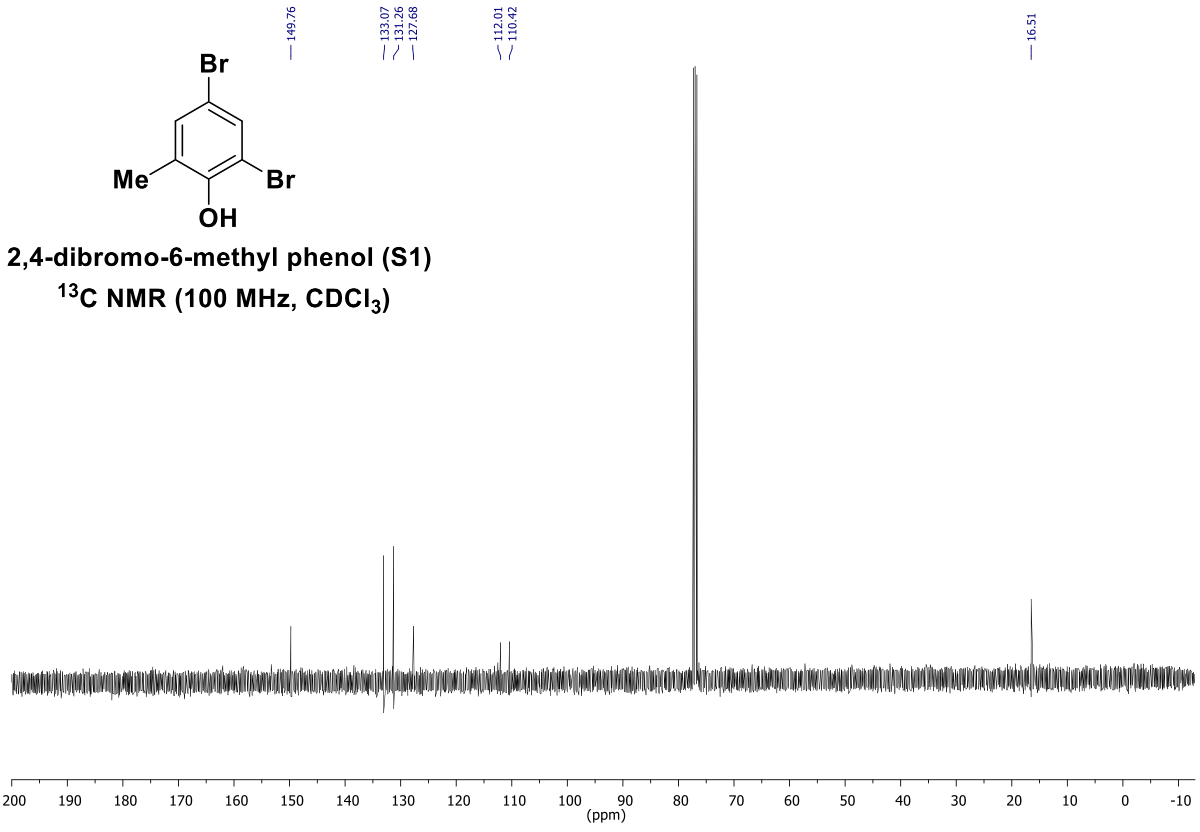

2,4-dibromo-6-methyl phenol (S1)

^{13}C NMR (100 MHz, $CDCl_3$)

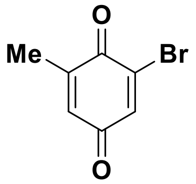

2-bromo-6-methyl-*p*-benzoquinone (S2)
^{1}H NMR (400 MHz, $CDCl_3$)

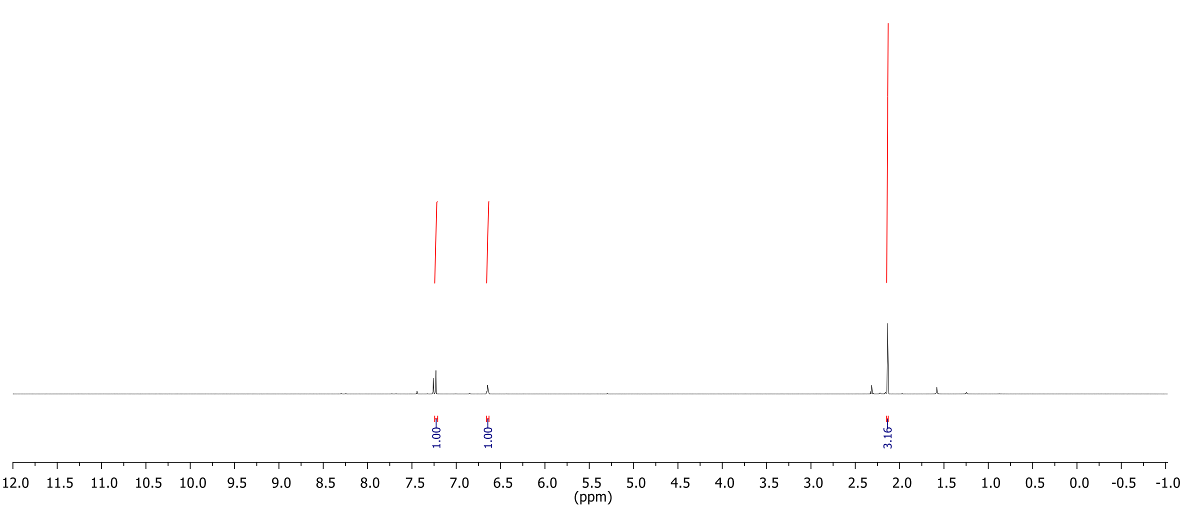

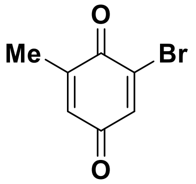

2-bromo-6-methyl-*p*-benzoquinone (S2)
^{13}C NMR (100 MHz, $CDCl_3$)

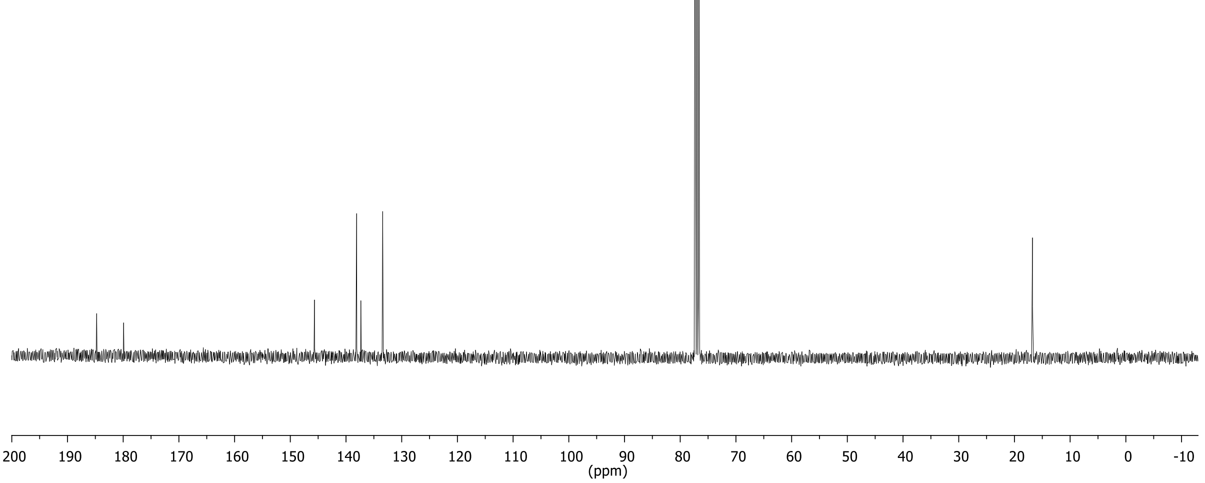

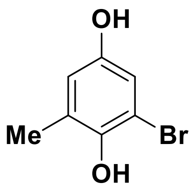

22

^{1}H NMR (400 MHz, CDCl$_3$)

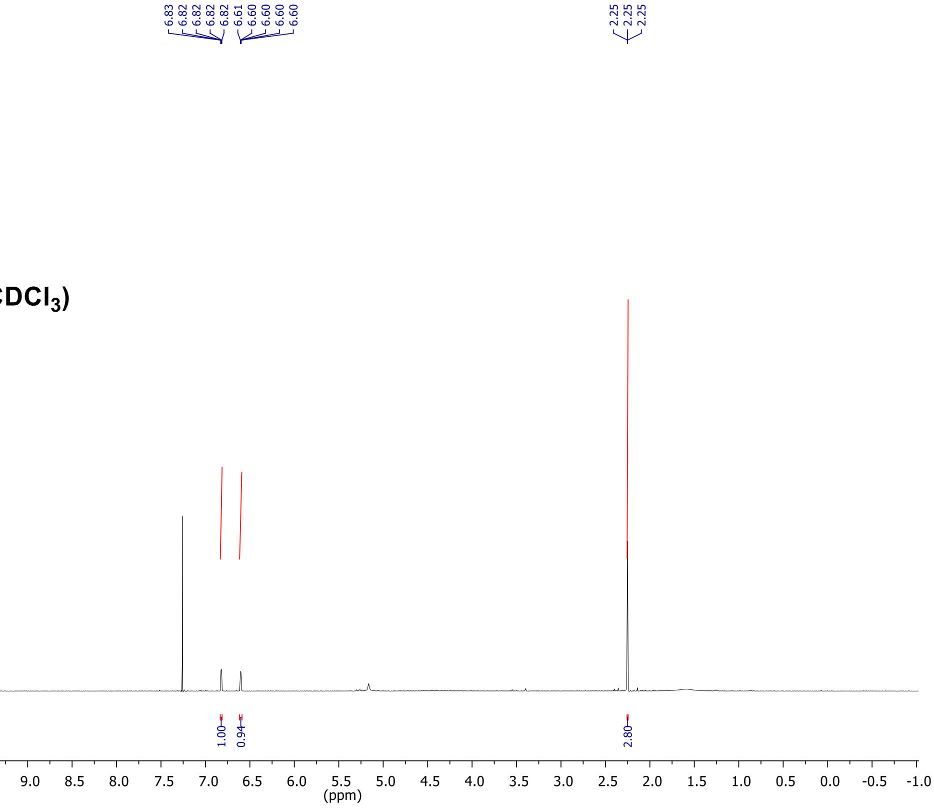

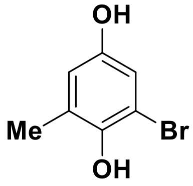

22

^{13}C NMR (100 MHz, CDCl$_3$)

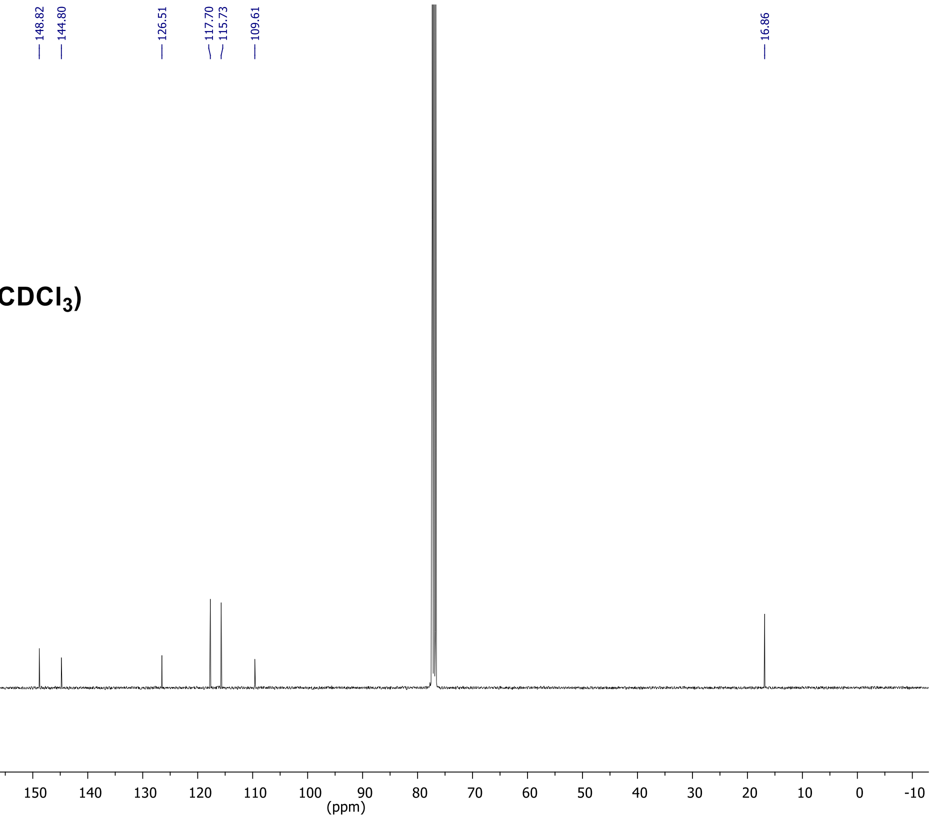

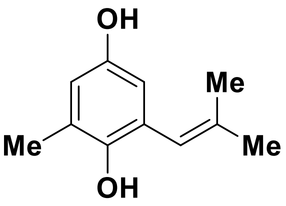

8

^{1}H NMR (400 MHz, CD_3OD)

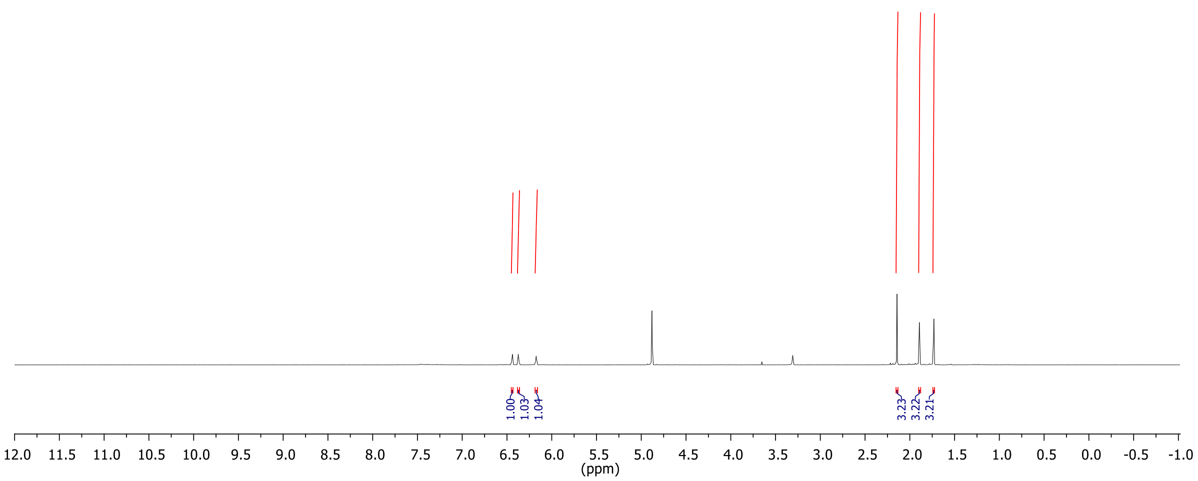

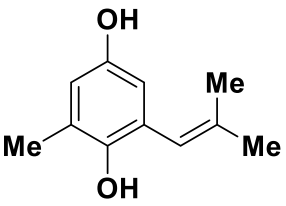

8

^{13}C NMR (100 MHz, CD_3OD)

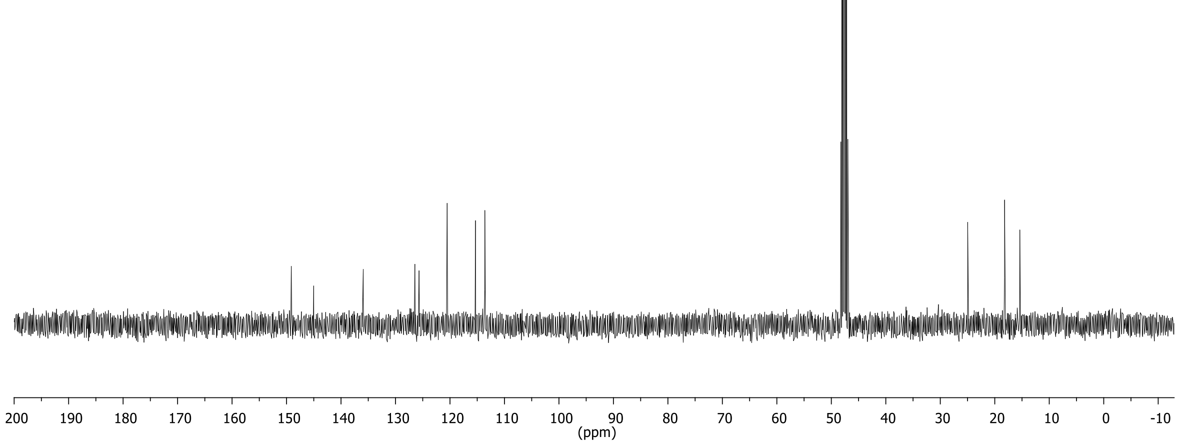

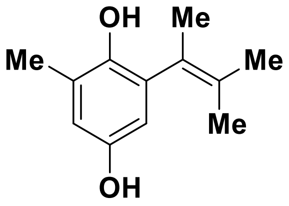

9

^{1}H NMR (400 MHz, $CDCl_3$)

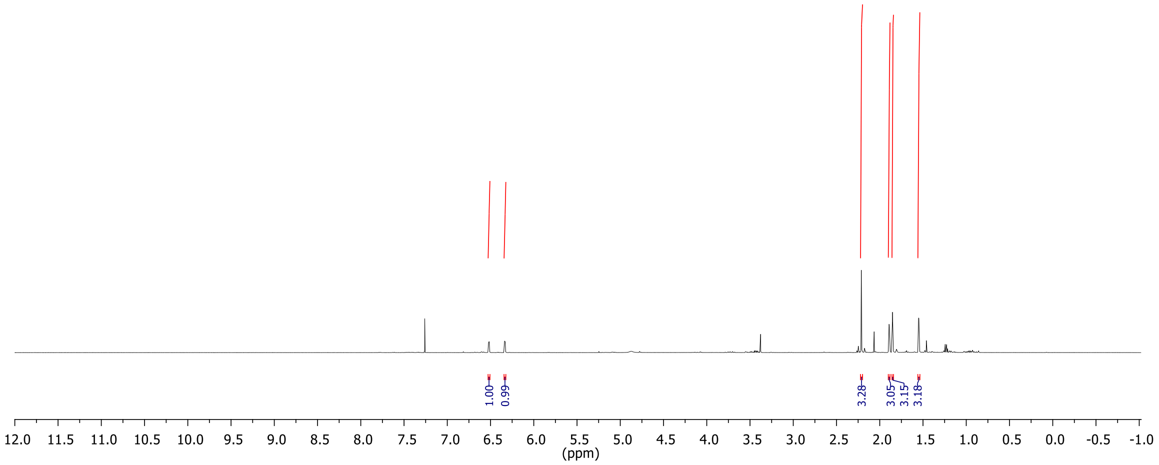

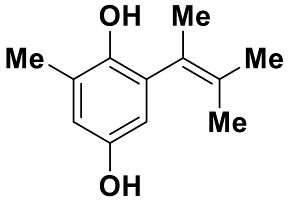

9

^{13}C NMR (100 MHz, $CDCl_3$)

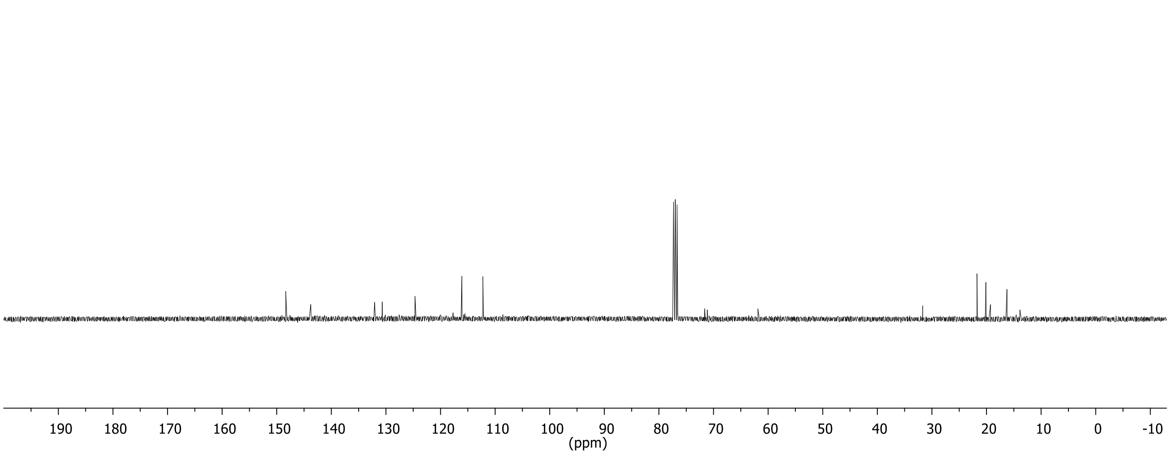

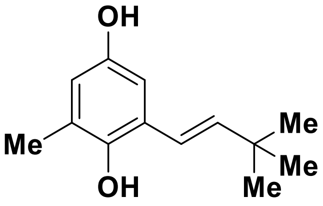

10

^{1}H NMR (400 MHz, $CDCl_3$)

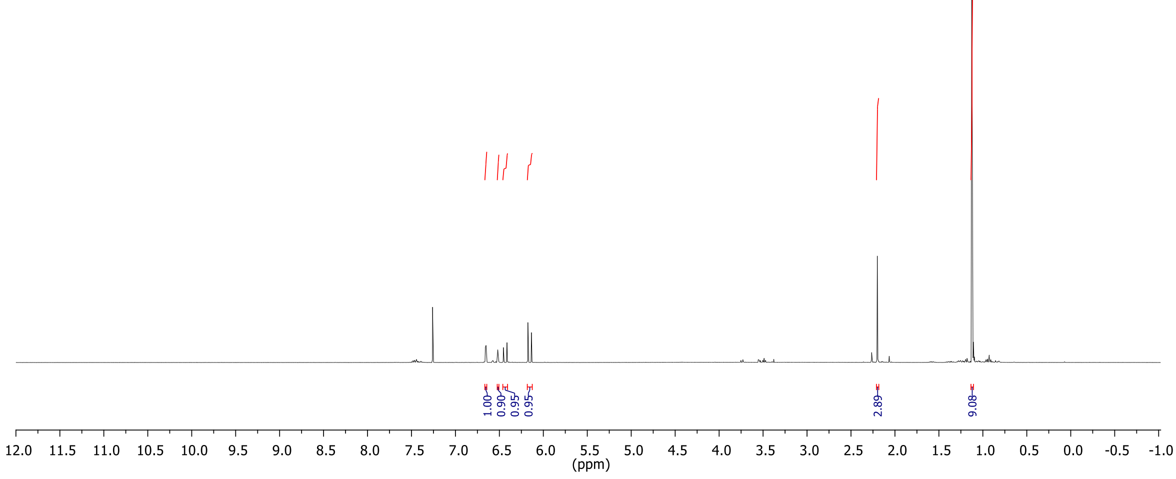

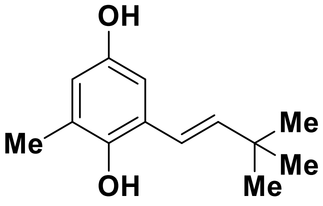

10

^{13}C NMR (100 MHz, $CDCl_3$)

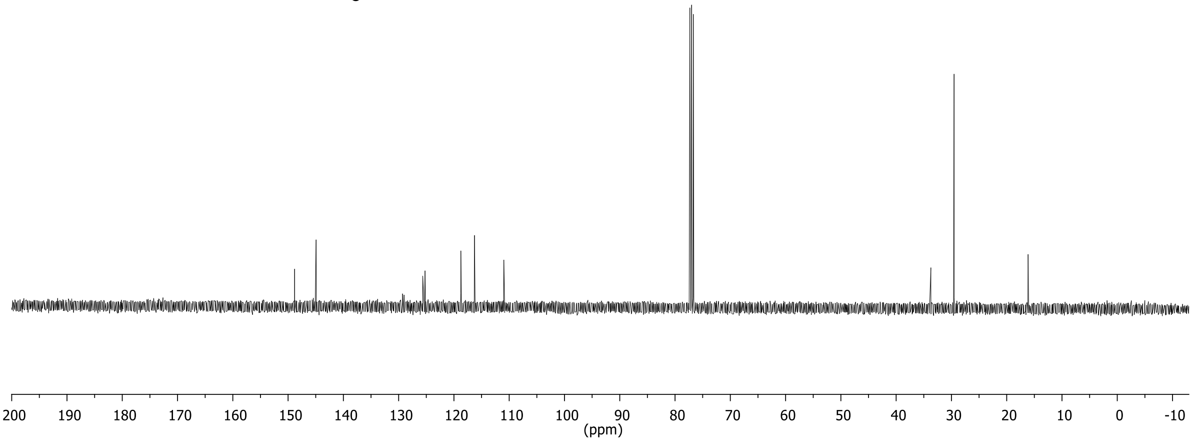

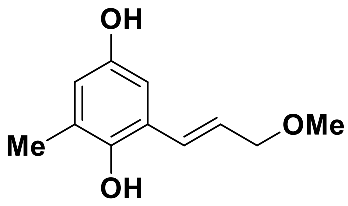

11

^{1}H NMR (400 MHz, CD_3OD)

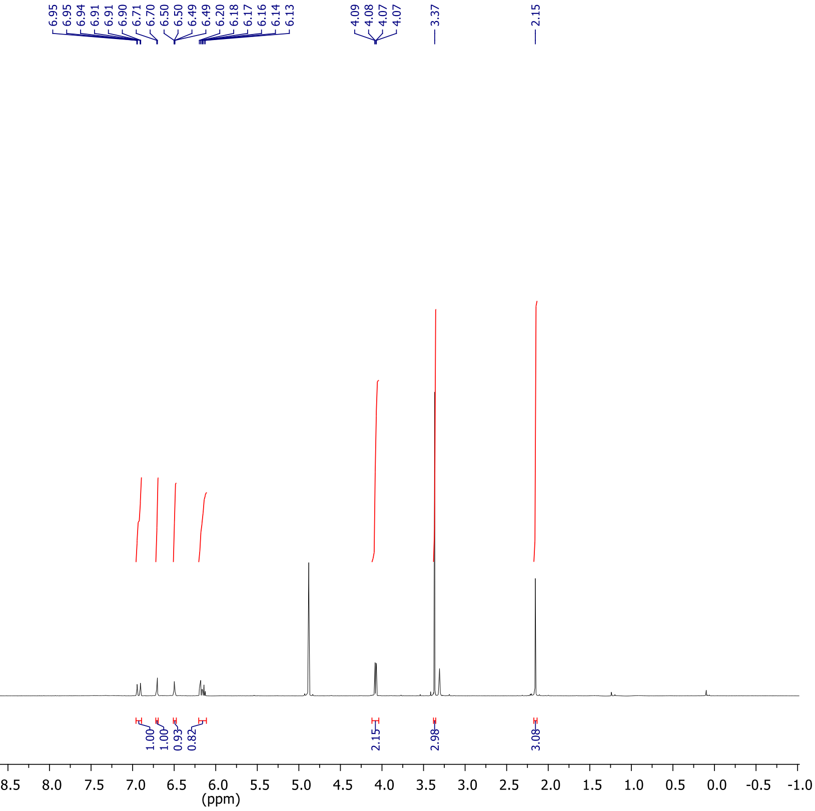

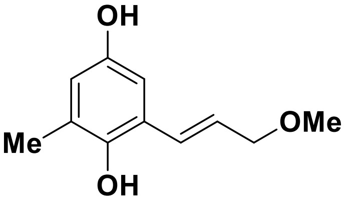

11

^{13}C NMR (100 MHz, CD_3OD)

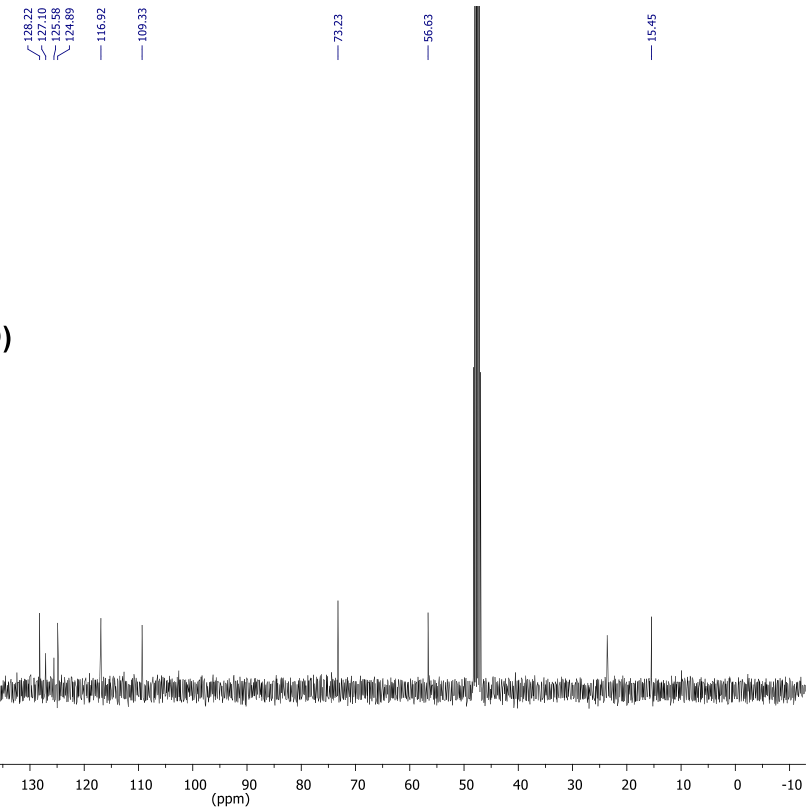

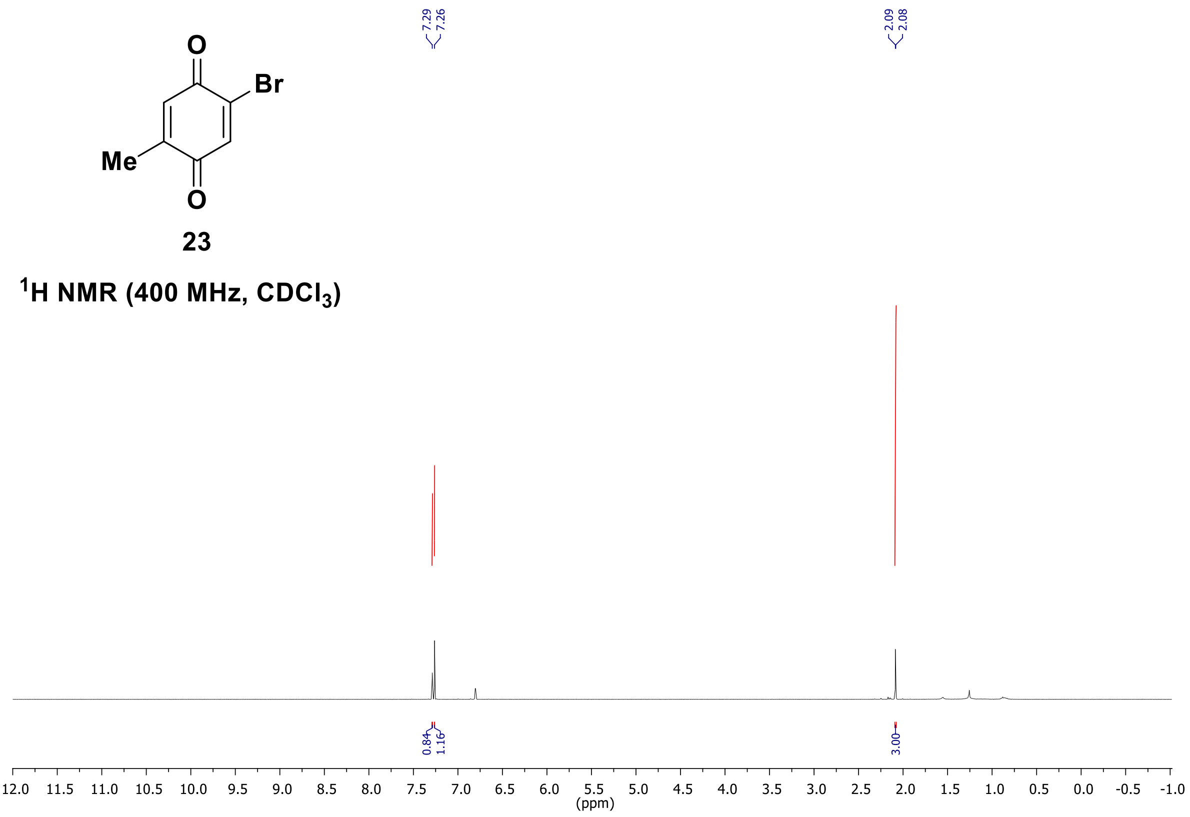
7.29
7.26
2.09
2.08
O
Br
Me
O
23
^{1}H NMR (400 MHz, $CDCl_3$)
0.84
1.16
3.00
12.0 11.5 11.0 10.5 10.0 9.5 9.0 8.5 8.0 7.5 7.0 6.5 6.0 5.5 5.0 4.5 4.0 3.5 3.0 2.5 2.0 1.5 1.0 0.5 0.0 -0.5 -1.0
(ppm)

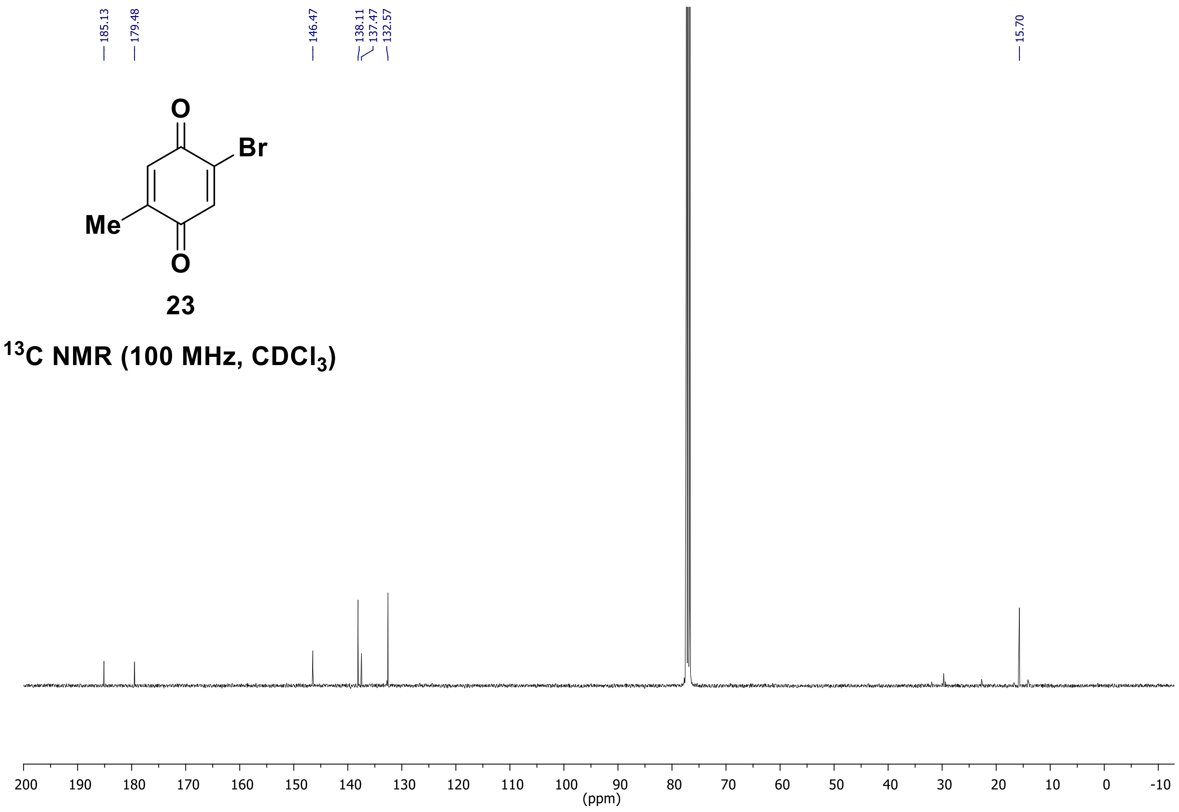
185.13
179.48
146.47
138.11
137.47
132.57
15.70
O
Br
Me
O
23
^{13}C NMR (100 MHz, $CDCl_3$)
200 190 180 170 160 150 140 130 120 110 100 90 80 70 60 50 40 30 20 10 0 -10
(ppm)

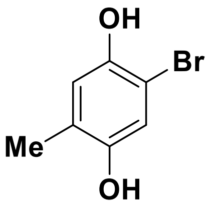

24

^{1}H NMR (400 MHz, CD_3OD)

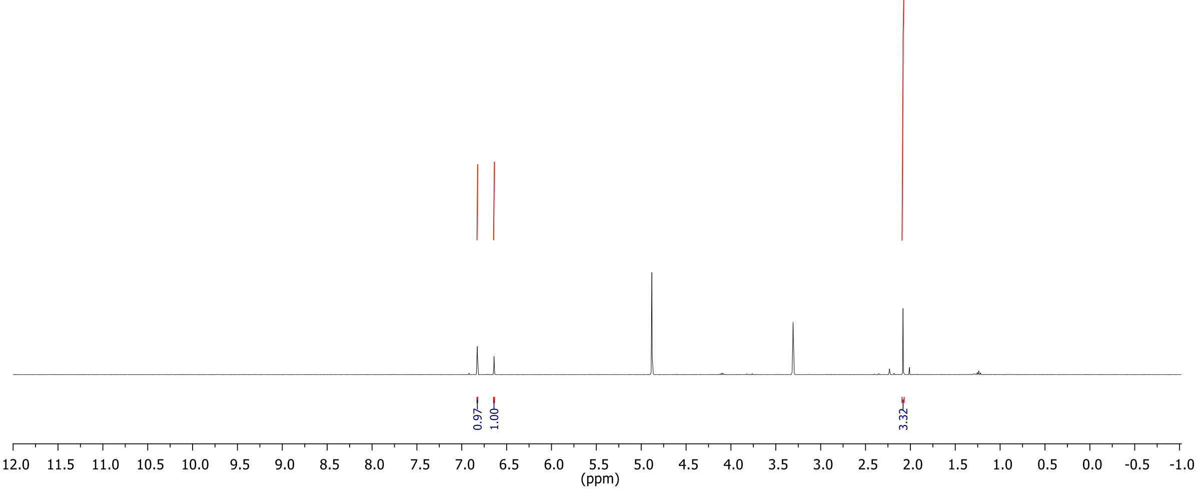

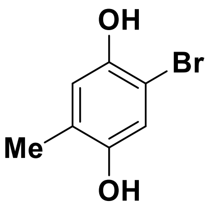

24

^{13}C NMR (100 MHz, CD_3OD)

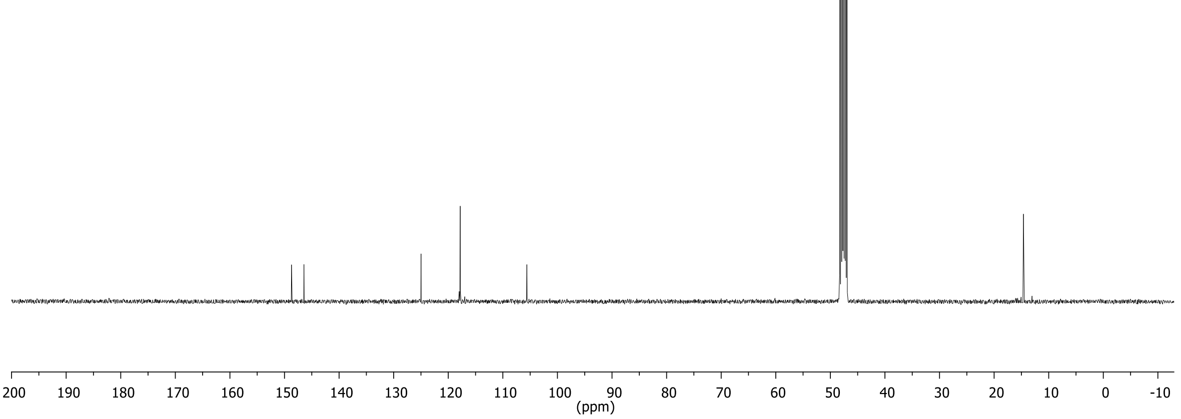

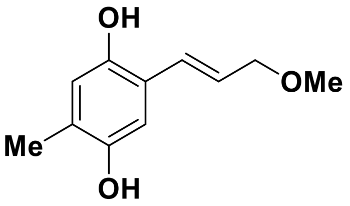

12

^{1}H NMR (400 MHz, CD_3OD)

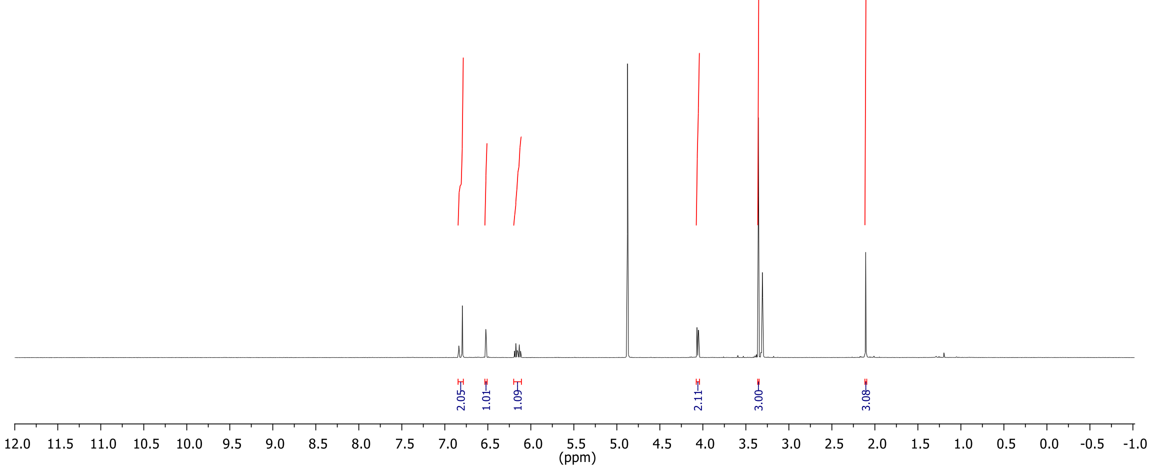

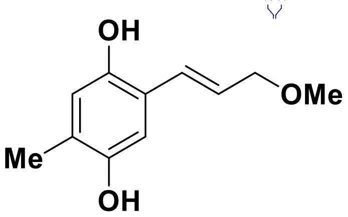

12

^{13}C NMR (100 MHz, CD_3OD)

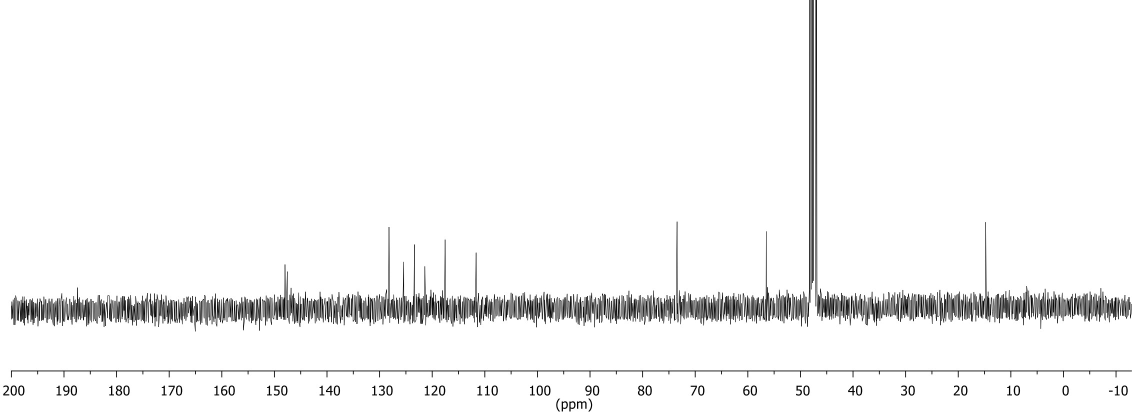

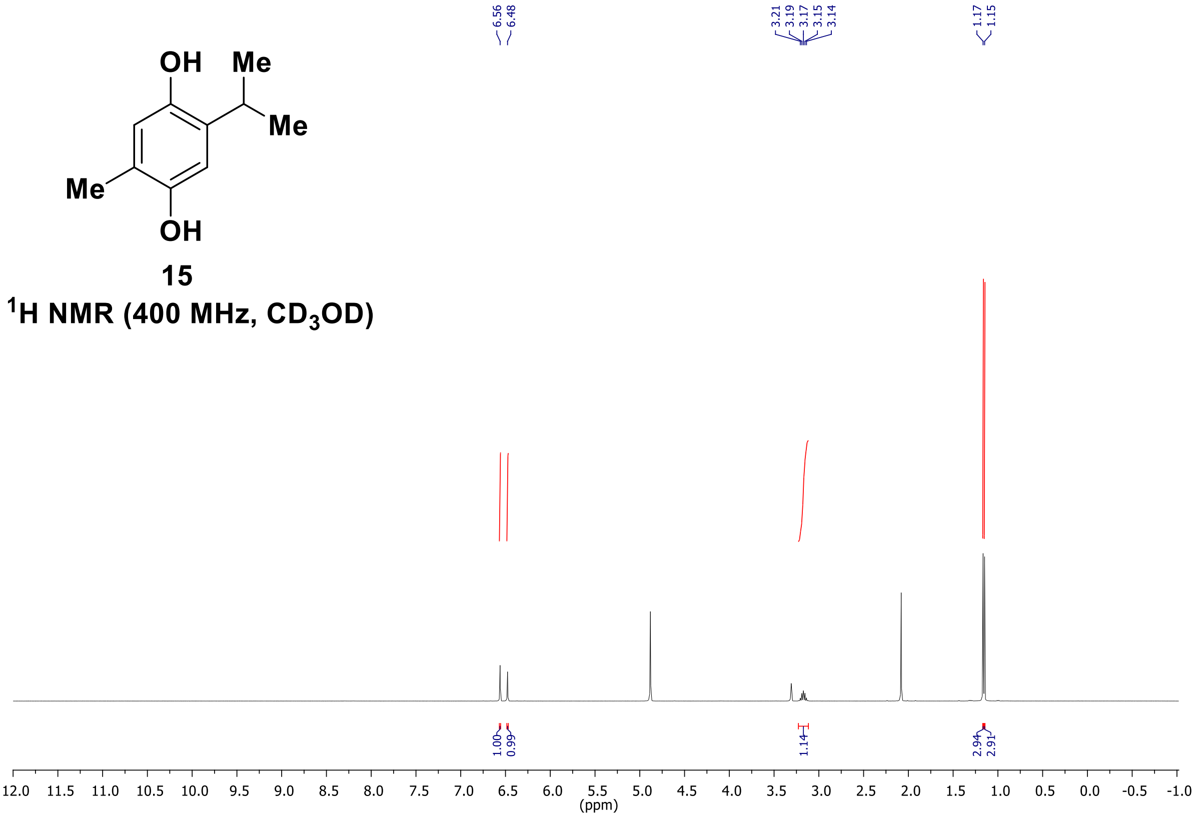

15

^{1}H NMR (400 MHz, CD_3OD)

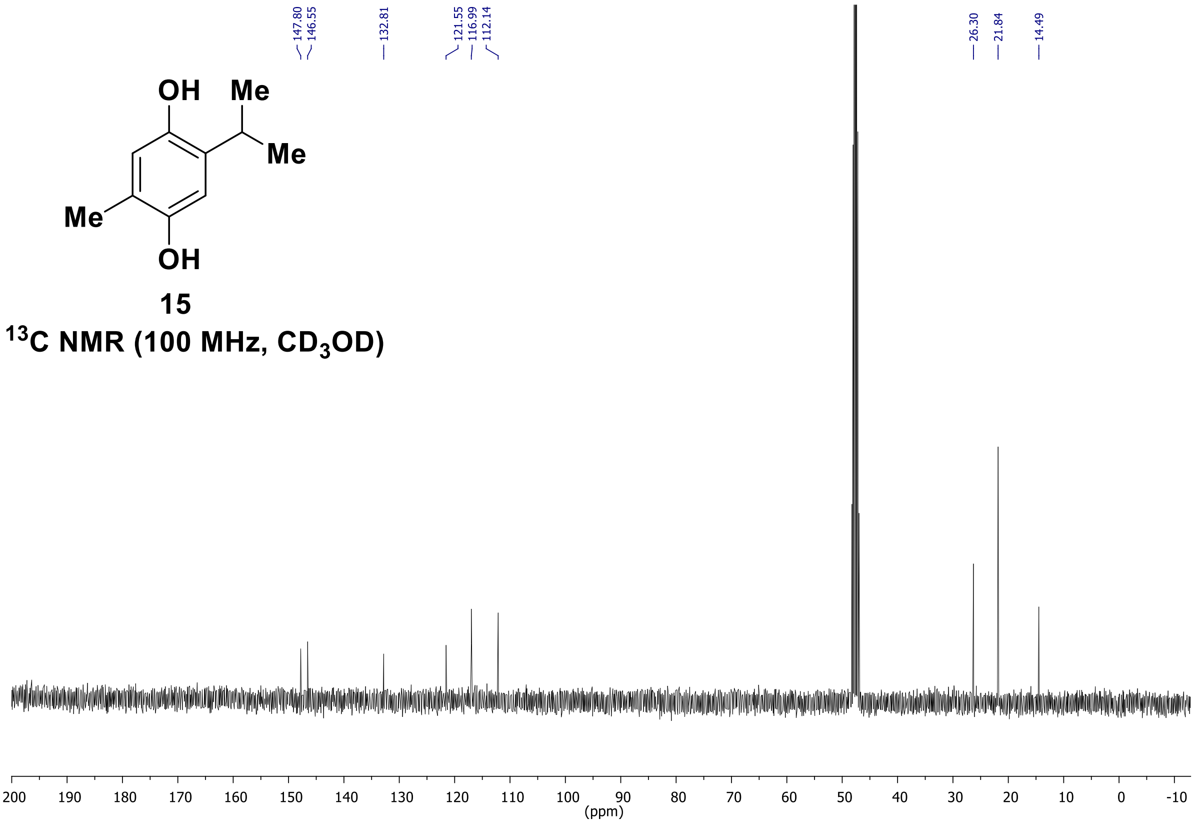

15

^{13}C NMR (100 MHz, CD_3OD)

Representative dose-response curves

Each point represents the mean of quadruplicates. SD values, typically lower than 10% of the mean values, have been omitted for clarity.

Toluquinol (compound **1**)

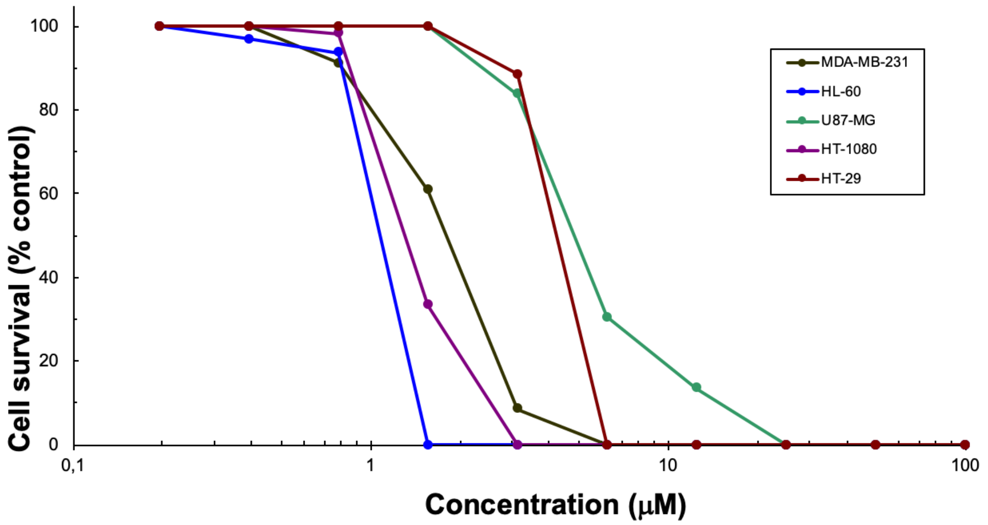

Compound **2**

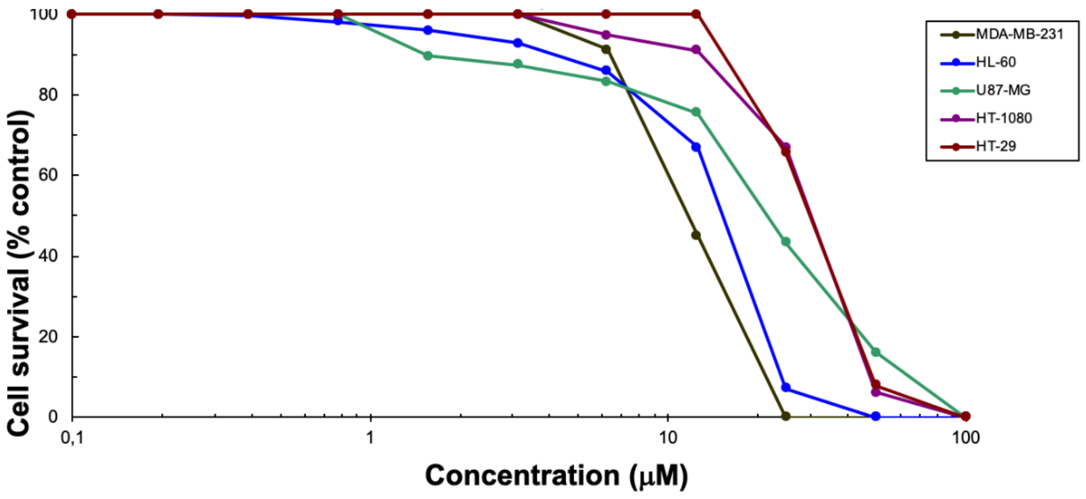

Compound **3**

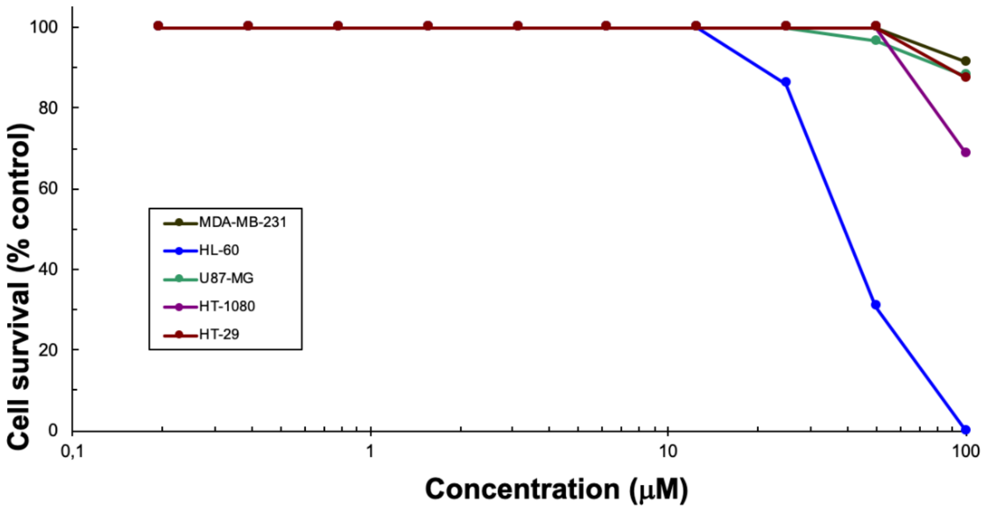

Compound **4**

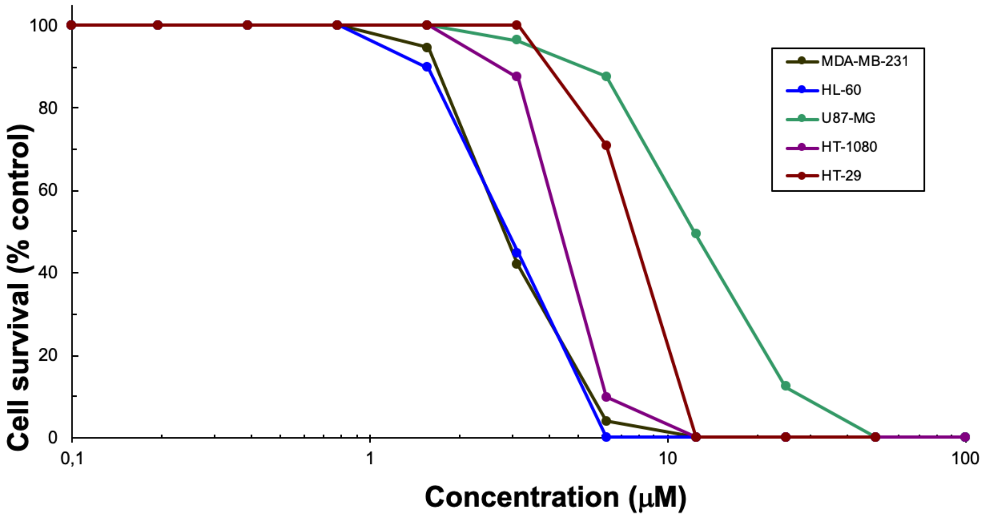

Compound **5**

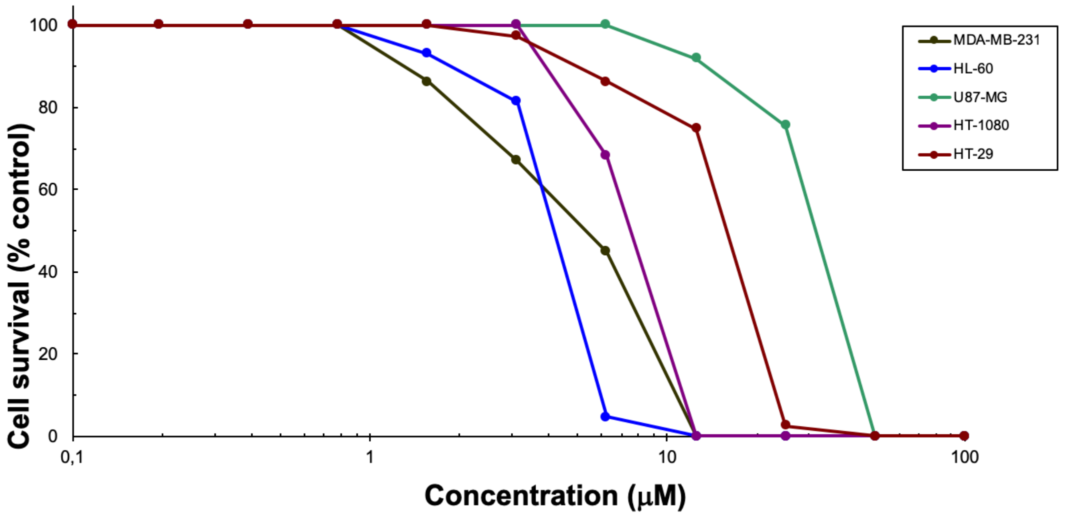

Compound **6**

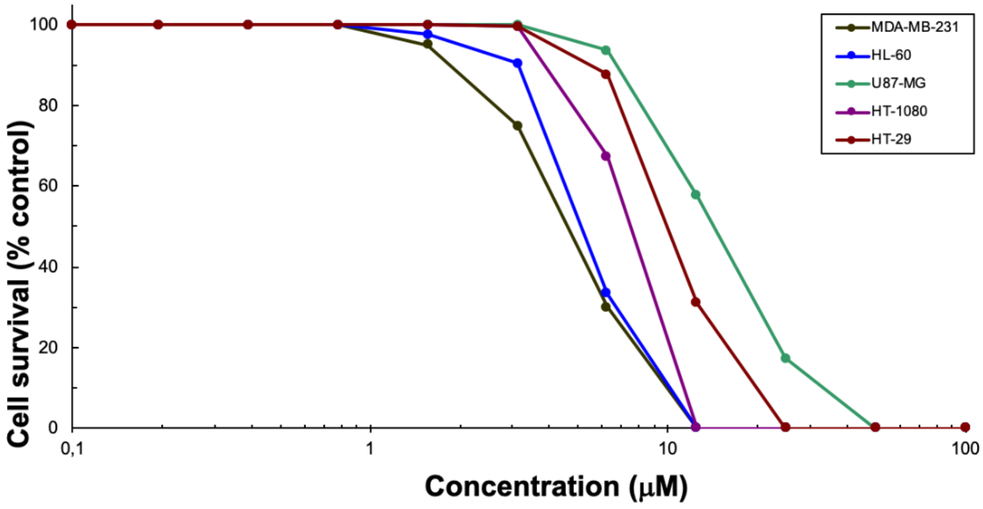

Compound **7**

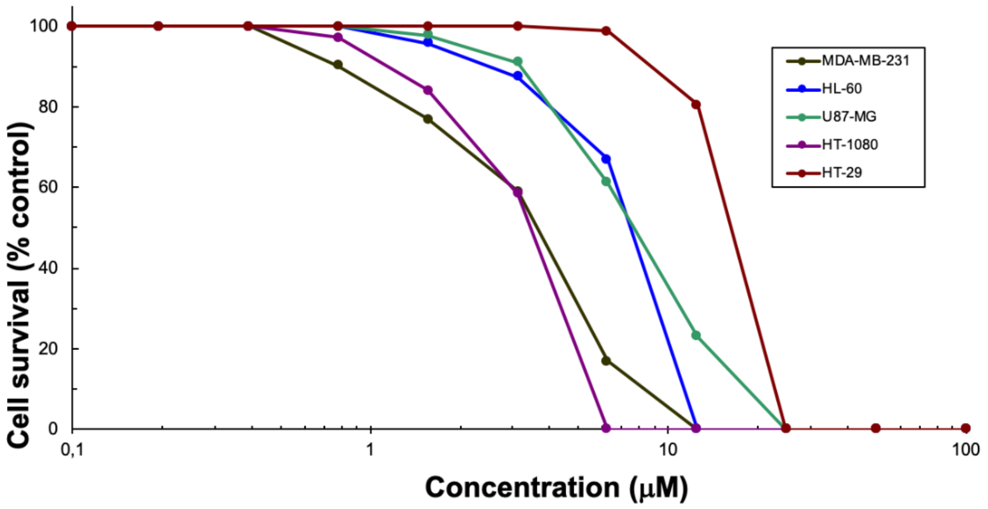

Compound **8**

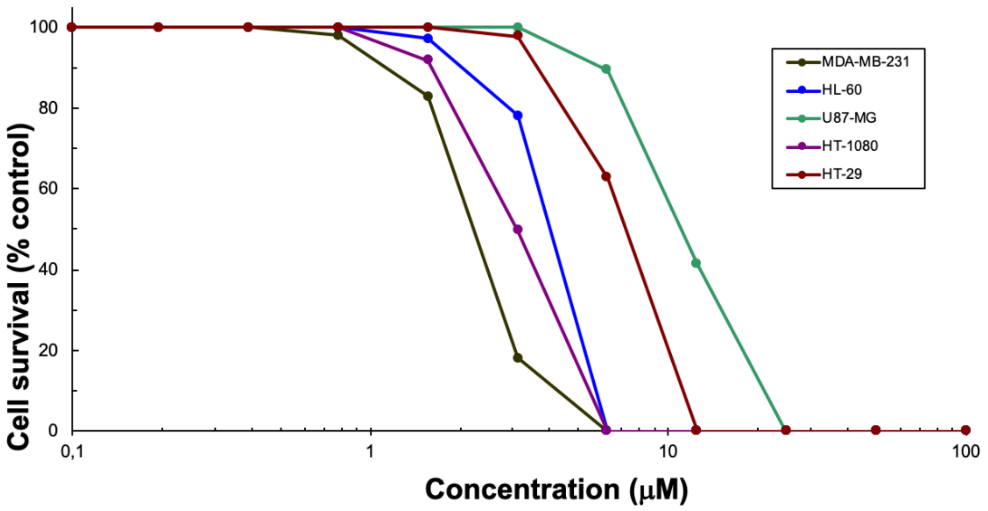

Compound **9**

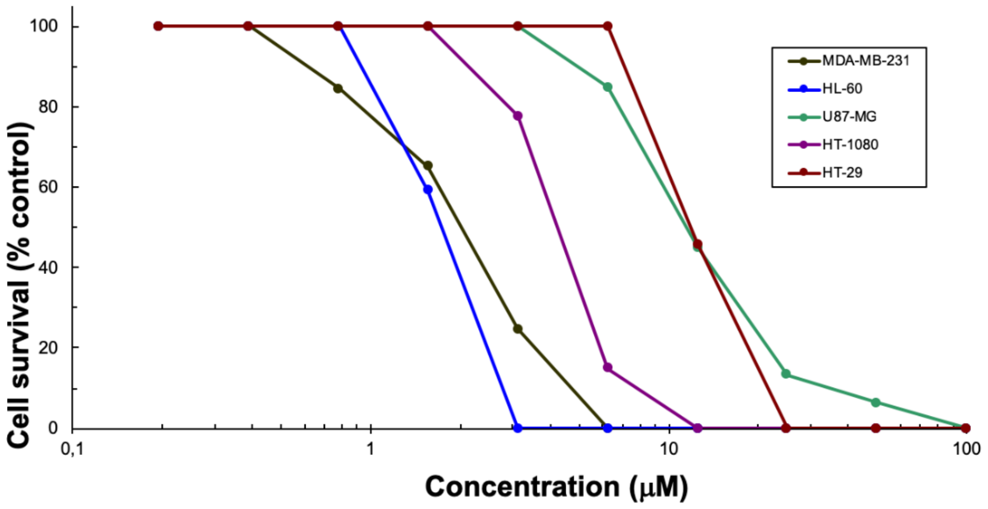

Compound **10**

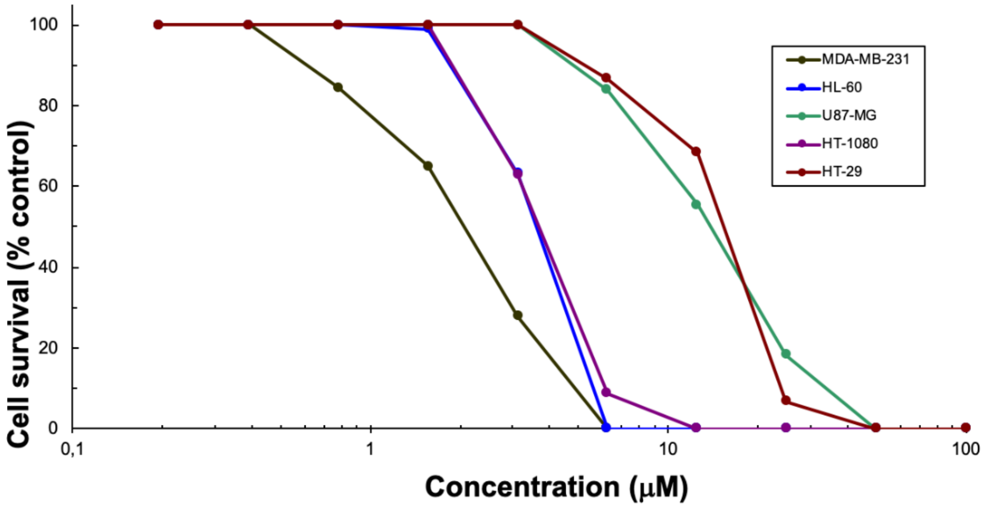

Compound **11**

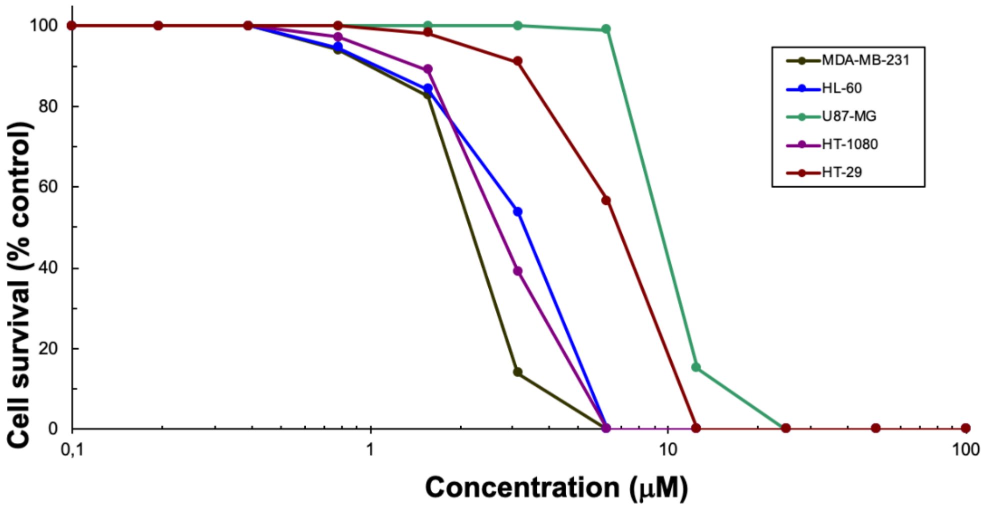

Compound **12**

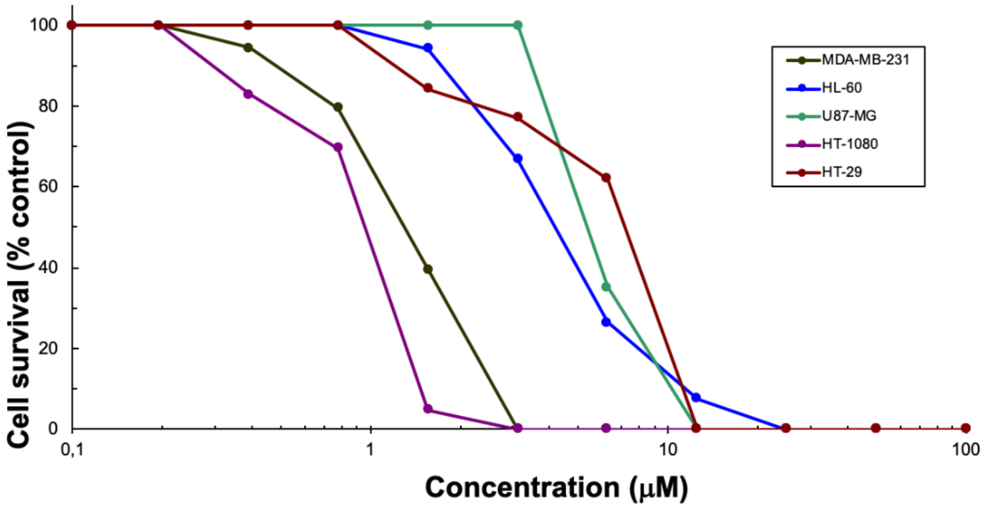

Compound **13**

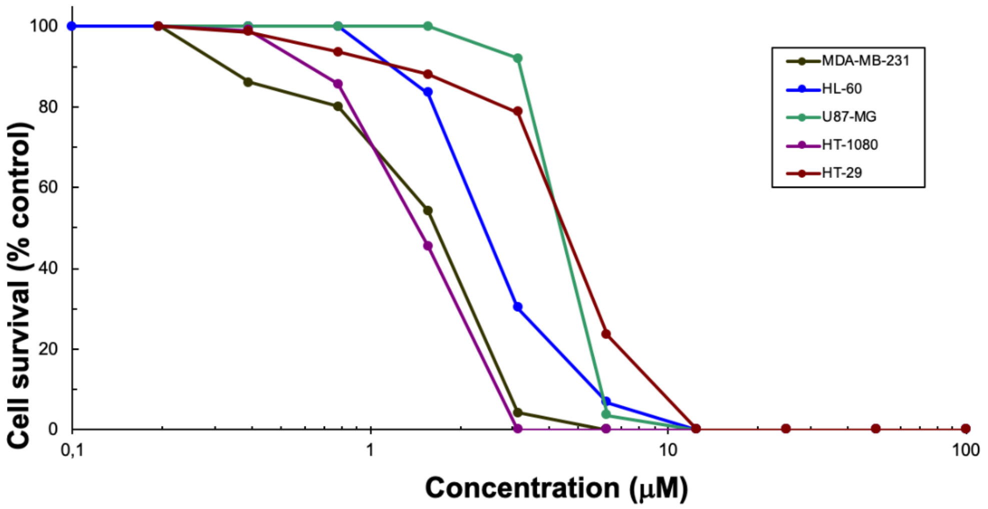

Toluquinone (Compound **14)**

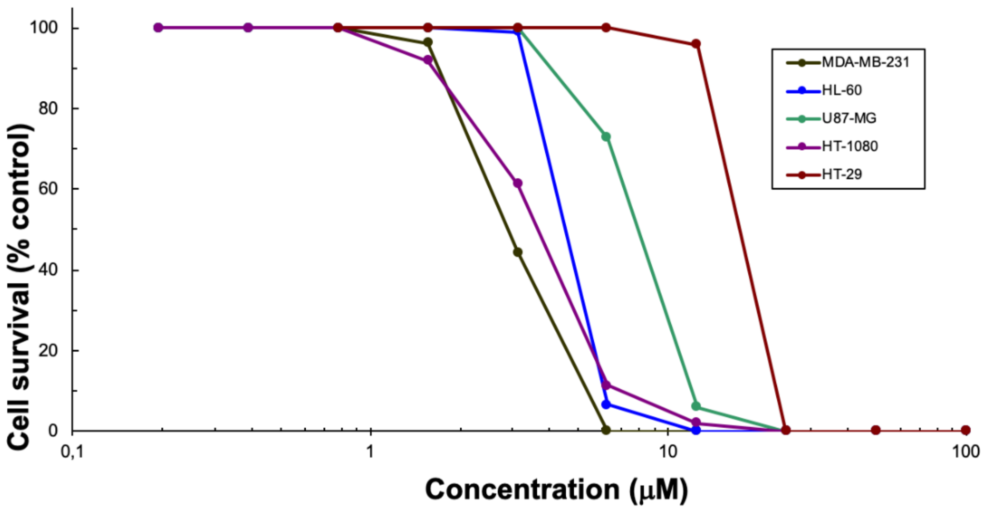

Thymoquinol (Compound **15**)

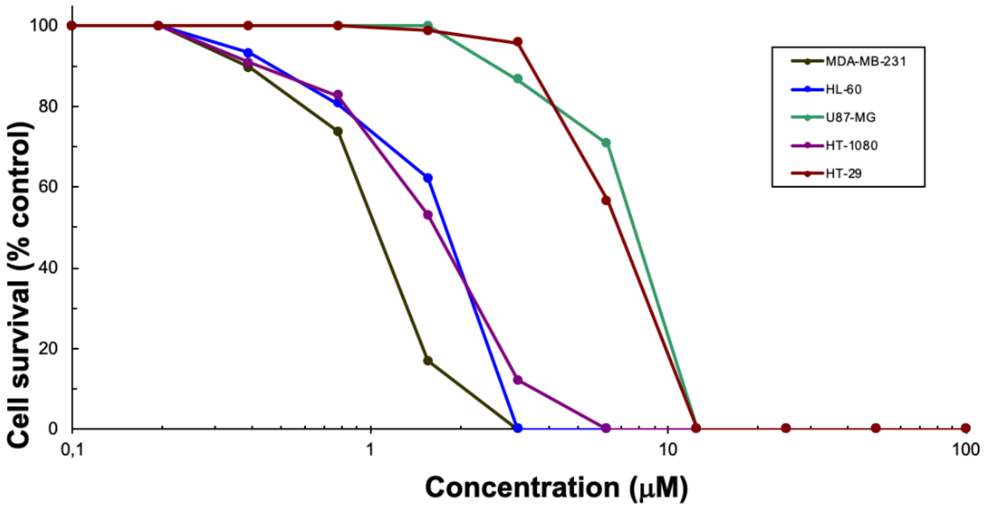

Thymoquinone (compound **16)**

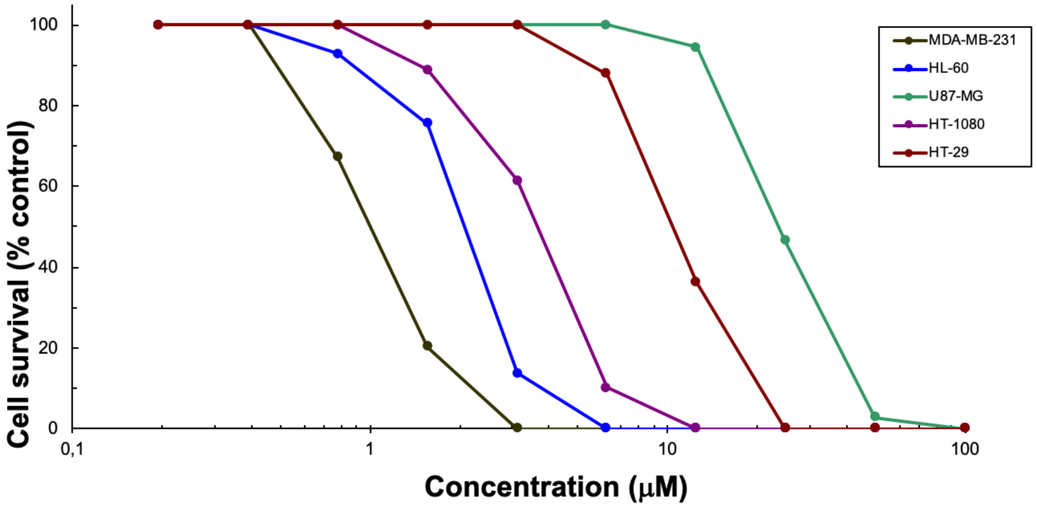

Compound **18**

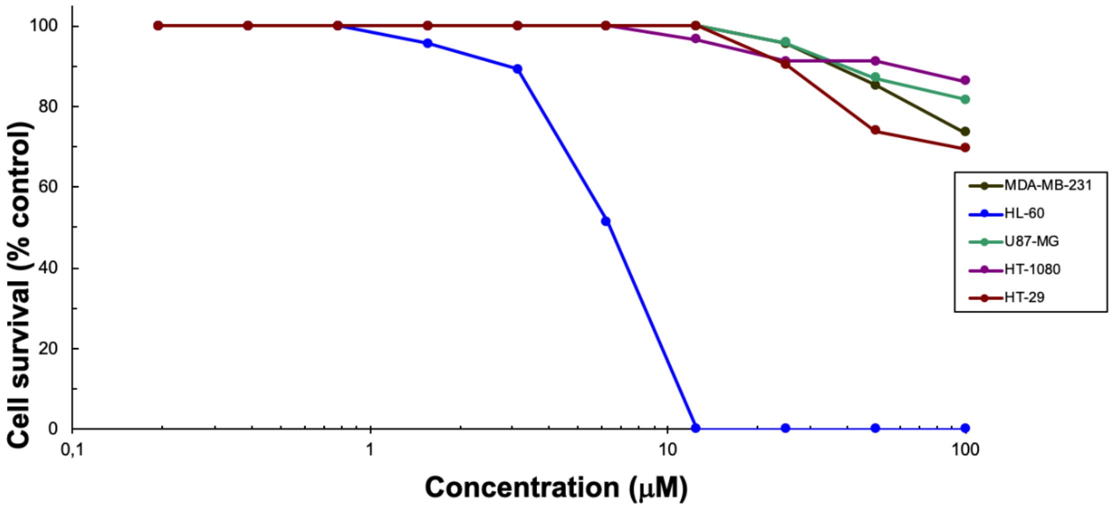

Compound **19**

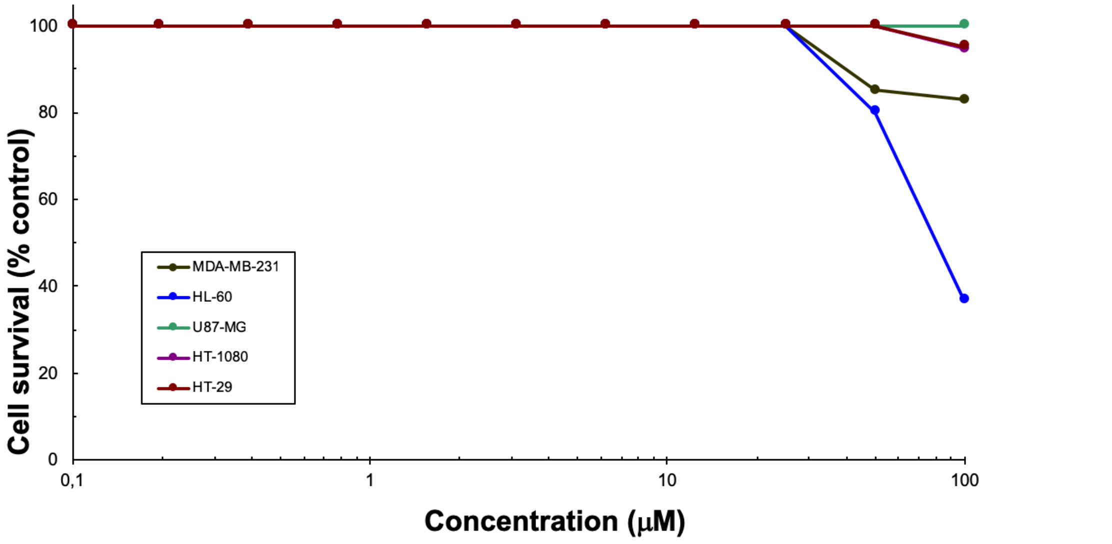

Supplement: Supplementary file 1 [file marinedrugs-17-00492-s001.pdf]
